# Structural Transition from Closed to Open for the Influenza A M2 Proton Channel as Observed by Proton-Detected Solid-State NMR

**DOI:** 10.1021/jacs.5c05111

**Published:** 2025-06-20

**Authors:** Swantje Mohr, Caspar Schattenberg, Tillmann Utesch, Henry Sawczyc, Veniamin Chevelkov, Sascha Lange, Jacek Kozuch, Han Sun, Adam Lange

**Affiliations:** † Research Unit Molecular Biophysics, Leibniz Forschungsinstitut für Molekulare Pharmakologie (FMP), 13125 Berlin, Germany; ‡ Research Unit Structural Chemistry and Computational Biophysics, Leibniz Forschungsinstitut für Molekulare Pharmakologie (FMP), 13125 Berlin, Germany; § Experimental Molecular Biophysics, Physics Department, 9166 Freie Universität Berlin, 14195 Berlin, Germany; ∥ Institut für Chemie, Strukturelle Chemische Biologie und Cheminformatik, Technische Universität Berlin, 10623 Berlin, Germany; ⊥ Institut für Biologie, Humboldt-Universität zu Berlin, 10115 Berlin, Germany

## Abstract

The influenza A M2 protein is an acid-activated proton channel and an established pharmaceutical target for antiflu drugs. Here, we studied the conductance domain of the tetrameric M2 channel (construct 18–60) using proton-detected solid-state NMR under native-like conditions in lipid bilayers. We obtained results at different pH values relevant to the virus life cycle: pH 7.8 (nonconducting, closed), pH 6.0 (opening), and pH 4.5 (conducting, fully open). In the closed state at pH 7.8, we detected two sets of resonances of the functionally important side chain of H37. Employing quantum mechanics/molecular mechanics (QM/MM) simulations, we assigned them to hydrogen-bonded and free H37 side chains occurring in varying ratios in the tetrameric arrangement. Additionally, some backbone signals also appear twice, suggesting conformational heterogeneity. The arrangement appears rather rigid, explaining the nonconducting nature of the channel. Lowering the pH to 6.0 leads to increased dynamics of the side chains, as manifested by their disappearance in CP based solid-state NMR spectra. This dynamic arrangement, which results from additional protonation of the four H37 side chains, allows for the efficient transport of protons through the channel. Finally, at pH 4.5, the conformational heterogeneity observed at higher pH values disappears completely, and a unique set of highly resolved resonances becomes visible. This suggests a well-defined acid-activated state of the M2 channel. Notably, in this state, the signals of the His37 side chains are absent due to dynamics, as well as the signals of the amphipathic helix (residues 45–52). This study provides strong evidence to a model of proton conduction through M2 which relies on dynamic vs rigid H37 side chains and furthermore lays the basis for an atomic structure of the acid-activated state of M2.

## Graphical abstract

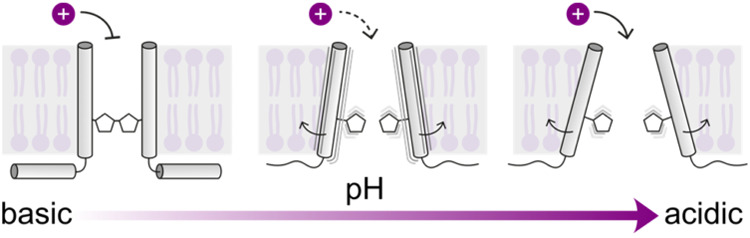

## Introduction

Viroporins are protein channels embedded within viral membranes that facilitate the transport of different molecules and ions. They are crucial for multiple steps of the viral life cycle, making them promising drug targets.^1,2^ As global climate change accelerates the spread of vector-borne viral diseases, such as West Nile and Dengue fever,^3,4^ understanding the structure and function of these viroporins is increasingly vital to future global health strategies.^1,5^

M2, a tetrameric proton channel from the influenza A virus, is the only viroporin so far that has been the target of licensed drugs, namely amantadine and rimantadine, both of which effectively block the wild-type of M2.^6^ Unfortunately, mutations such as S31N, which are prevalent in circulating virus strains,^6^ have conferred resistance to these drugs.^7^ Nevertheless, M2′s simple structure, consisting of only one α-helix spanning the membrane, makes it a well-established and widely studied model system for viroporins in general and proton channels in particular.

Despite its simplistic architecture, the M2 channel plays a crucial role in the infection process of the influenza virus. Upon entry into an infected cell via endocytosis, the virus is exposed to a highly acidic environment (Figure 1A). This acidic milieu activates the M2 channel, allowing protons into the viral capsid thus facilitating a decrease in pH within the viral particle, which subsequently triggers the release of viral RNA into the host cell. Furthermore, M2 has been shown to be involved in the budding process of newly formed viral particles.^8^

**Figure 1:**
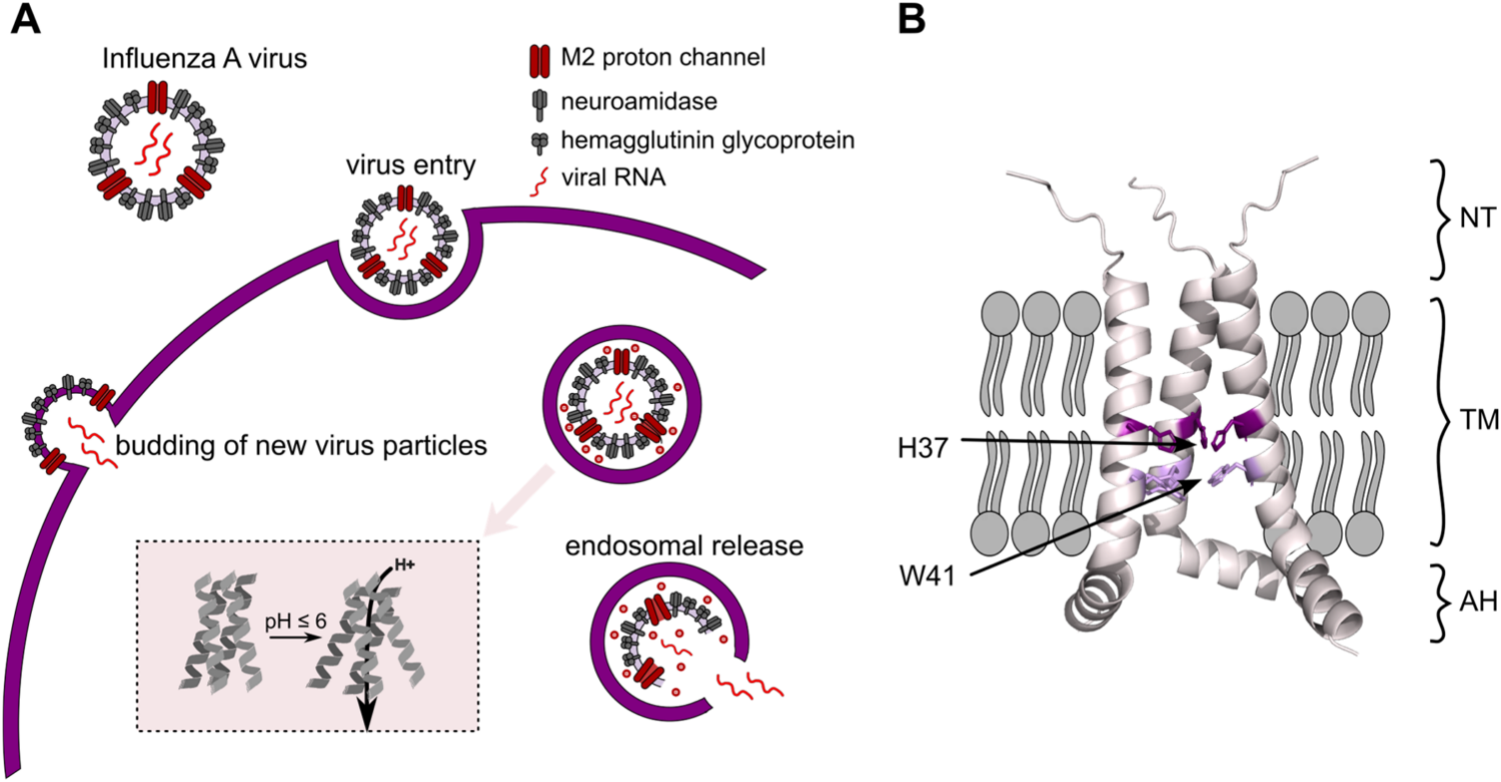
(A) Representation of the influenza lifecycle, noting M2′s functional roles, starting with its activation in the low-pH endosome, leading to opening of the virus particle and finally the release of viral RNA into the host cell and the budding process of new viral particles at the end of the cycle. Inset: Scheme of acid activation of M2 transmembrane domain (TM). (B) Structural properties of the M2 conduction domain (res. 18–60), shown on an AlphaFold2 model, with the N-terminal part (res. 18–21, NT), the TM domain (res. 22–46) and the amphipathic helix (res. 47–60, AH). Residues reported to be involved in the conduction mechanism are highlighted in purple.

M2 is a 97-residue membrane protein, consisting of a highly conserved, unstructured N-terminal domain (residues 1–21) involved in the incorporation of the protein into the virion and the conformational equilibrium of the channel^9,10^ (Figure 1B). The transmembrane domain (TM, residues 22–46) regulates the conduction mechanism and contains a tryptophan residue as a substrate gate.^11^ Following this, an amphipathic helix (AH, residues 47–62) and the C-terminus (residues 63–97) contribute to proton conduction,^12^ facilitate interactions with other viral proteins, and enable virion assembly and budding.^13,14^

While only a few studies have structurally characterized the full-length M2 protein,^15–18^ extensive research has been conducted on its TM domain alone or together with the adjacent AH – together referred to as the conduction domain (CD). While the TM domain alone is able to conduct protons,^19^ this region is considered critical for native-like conduction rates and has been investigated using various biophysical techniques.^20^ Most published structures of M2TM and M2CD, derived from both solution and solid-state NMR, as well as X-ray crystallography in different environments—including detergent micelles, lipid cubic phase, proteoliposomes with simple and viral mimetic lipid mixtures—reveal a homotetrameric arrangement.^21^ Focusing specifically on M2CD (constructs including residues from ∼18 to ∼62), the first structural insights came in 2010 from Cross and co-workers, who used orientated sample (OS) solid-state NMR at a neutral pH of 7.5.^22^ Their findings showed a symmetric structure, with symmetry breaking occurring only at the histidine residue in the TM domain. In this configuration, the histidine tetrad adopts a +2-protonation state, forming a locked conformation where two histidines establish an intermolecular hydrogen bond with the adjacent monomer, while another intramolecular N_δ1_–H–O hydrogen bond of the same histidine stabilizes the structure. Subsequent studies incorporating magic-angle spinning (MAS) solid-state NMR at a pH of 6 observed peak doubling exclusively for H37 and W41, both of which are involved in this structural hydrogen bonding, but the overall structure of the pore remained homotetrameric.^23,24^ Multiple additional studies focusing on different aspects such as drug binding,^25^ interactions with the membrane,^26–28^ and hydrogen bonds in full-length constructs reinforced the homotetrameric framework.^29,30^

However, recent solid-state NMR studies have challenged the previously accepted symmetric conformation, instead suggesting a dimer-of-dimer fold not only for the histidine tetrad but also for the entire TM domain instead.^31–33^ Initial analysis of the wild-type construct at pH 7.8 in DPhPC proteoliposomes, later extended to the drug-resistant S31N mutant, for which then a structure was determined (PDB ID: 2N70), and other lipid mixtures and ratios,^32^ revealed peak doublings for all transmembrane residues. This indicated a structural arrangement in which adjacent monomers are shifted relative to their neighbors. Additionally, the hydrogen bonding between histidine residues for the nonconducting arrangement, as previously described,^34,35^ was observed again at a pH of 7.8. This study also highlighted the role of bound water molecules,^36^ prompting renewed discussion about the proton conduction mechanism in the M2 channel.

Currently, two competing models exist for proton conduction in the M2. The first model involves low-barrier hydrogen bonds (LBHB) between H37 residues, forming imidazolium-imidazole pairs that facilitate proton transfer via a transient +3 state.^23,24,29,37^ The second model by Hong and co-workers proposes a water-mediated shuttling mechanism, where H37 exchanges protons without intermonomer hydrogen bonds. Instead, hydrogen bonds only exist between water molecules and histidine side chains, and protons are shuttled through the pore facilitated by imidazole ring reorientation.^38–41^

Besides these experimental findings, computational methods have also contributed to the functional understanding of M2. Here, the effect of hydration, conformational changes, and proton permeation were investigated in classical molecular (MD) dynamics^41,42^ , multiscale,^40,43,44^ and constant pH simulations.^45,46^

To further advance the understanding of the M2 channel, we present here a study employing proton-detected ultrafast magic-angle spinning (MAS) solid-state NMR, a method already proven to be exceptionally effective when studying small membrane proteins at atomic resolution,^47–50^ to characterize M2CD for the first time at three different functionally relevant pH conditions. This extends the previous proton-detected solid-state NMR investigation of M2CD at pH 7.8 by L. Andreas, R. Griffin and co-workers.^31–33^ Complemented by functional studies, atomistic molecular dynamics (MD) simulations, quantum mechanics/molecular mechanics (QM/MM) calculations, and density functional theory (DFT) calculations of the chemical shifts, our analysis reveals significant structural differences at atomic scale across different pH levels. This integrated approach, being successful in multiple problems in the past,^51^ here also provides unprecedented insights into the pore opening process of the M2 channel, representing a critical step forward in elucidating its activation mechanism.

## Results and Discussion

### Homotetrameric Structure in a High-pH Environment

Starting with a sample reconstituted into -lipid proteoliposomes at a pH of 7.8, where the channel is thought to be in a closed form, we were able to assign all of the residues of the TM domain and the AH of M2 (Figure 2A, purple spectrum, and Table S1).

**Figure 2:**
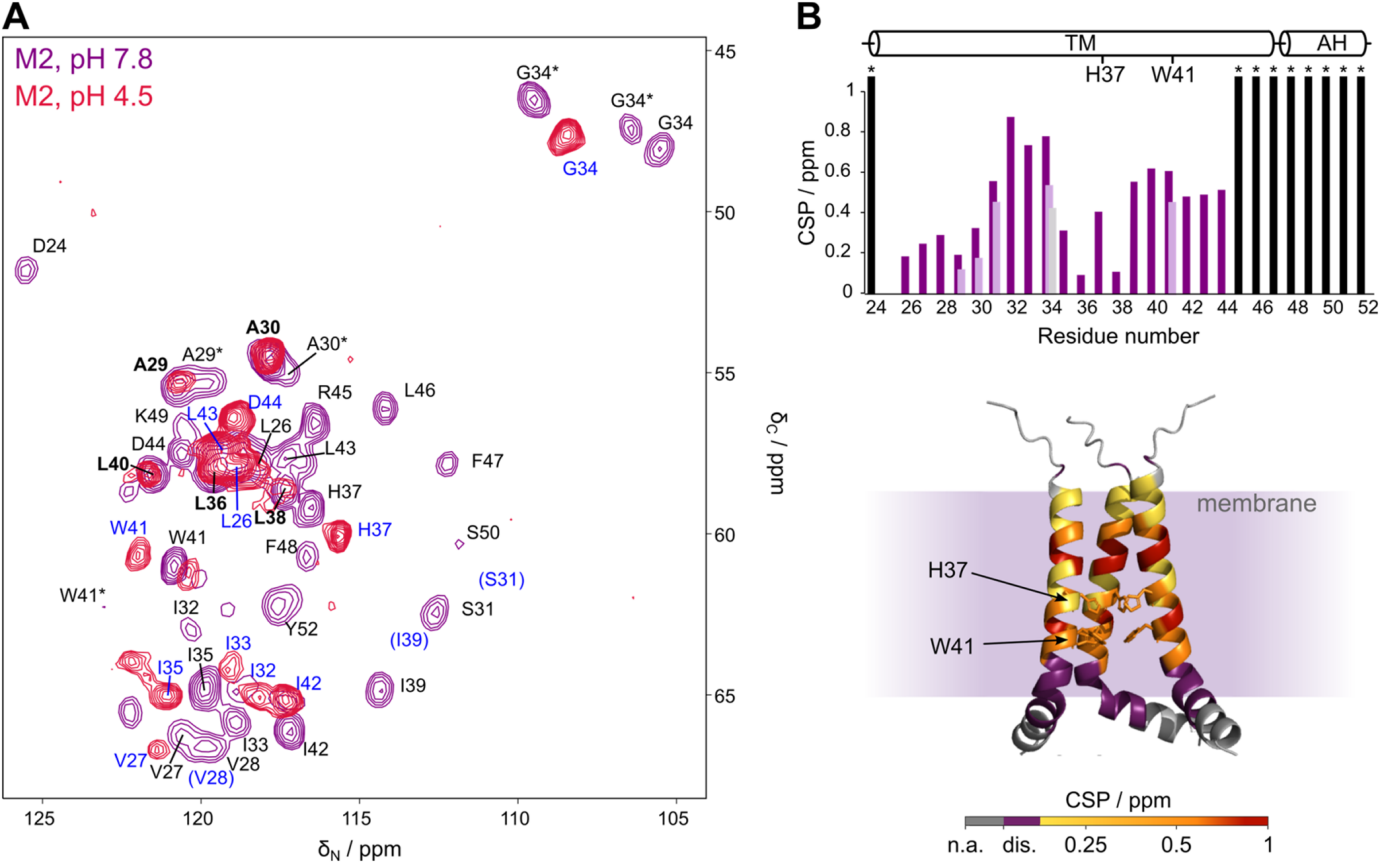
Structural changes of M2 at pH 7.8 and pH 4.5. (A) NC-projections of (H)­CαNH 3D spectra of the conduction domain of M2 at pH 7.8 (purple) and pH 4.5 (red). Assignments are marked in black for the pH 7.8 conformation, blue for the pH 4.5 conformation and bold if the residue shows the same chemical shift in both pH conditions. Doubled or tripled peaks occurring at pH 7.8 are marked with an asterisk (*). Chemical shift perturbations (CSP) are shown in (B) on the top. Residues showing multiple resonances at pH 7.8 are displayed with multiple bars, showing different CSP values for each peak. Disappearing resonances are shown in black and marked with an asterisk (*). On the bottom, CSP is color-coded and applied on an AlphaFold2 structure of the CD of M2, with unassigned residues (n.a.) marked in gray, and disappearing resonances (dis.) shown in dark purple. Only 3 monomers of the tetrameric channel are shown.

In contrast to previous studies of M2 S31N (18–60) in DPhPC lipids,^31^ later extended to other lipid mixtures,^32^ peak doubling is only observed for the residues G34, W41, A29, and A30, indicating a predominantly homotetrameric structure of the channel, which is only slightly interrupted near the N-terminal part of the TM domain and the expected substrate gate W41. For G34, the detection of even three distinct signals supports the hypothesis of structural heterogeneity rather than a strict 2-fold symmetry. Additionally, peak intensities deviate significantly from a 1:1 ratio between the two conformations (for example, W41/W41* in Figure 2A), which would be expected in a dimer-of-dimers structure for the channel. This structural heterogeneity could arise from multiple factors, including helix bending around the glycine residue as well as imperfect helix packing within the membrane as previously discussed for other M2 constructs.^29^ Additionally, interactions with the membrane, although so far only implicated by studies in viral-mimetic lipid membranes on clustering^28,52^ and cholesterol binding,^26^ could still contribute to heterogeneous channels.

Focusing on the histidine, heterogeneity could also be caused by different protonation states of the side chain: While some studies report the protonation of the first histidine to occur at pH values >8,^37^ others determined its p*K*_a_ to be lower than 7.5,^29,53,54^ putting our sample condition in a range where transition from one state to the other could cause heterogeneous structures.

This phenomenon was already discussed in a previous study from our lab,^54^ where the same construct reconstituted in a DPhPC membrane showed peak doubling for a different set of residues. Given the otherwise similar sample preparation, this suggests that the membrane environment plays a significant role in structural heterogeneity.

### Histidine Hydrogen Bonding Network at pH 7.8

Apart from the backbone structure, H37 is of special interest, as it could potentially form a hydrogen-bonded tetrad with its neighboring histidines from adjacent subunits (see Figures 3 and 4), as suggested by previous studies.^24,34,35^ In this high-pH environment, we detected N–H signals in both cross-polarization-based (CP, through space) as well as J-coupling-based (INEPT, through bond) (H)­NH spectra (Figure 4, first panel) and H–C cross-peaks in CP-based experiments (Figure S2). The simultaneous observation of H37 signals in both CP and INEPT suggests that the imidazole side chains are in an intermediate motional regime. They are sufficiently rigid to retain dipolar couplings for CP transfer yet exhibit enough local motion to allow for INEPT signal buildup under our experimental conditions. Notably, the CP and INEPT (H)­NH spectra as well as the CP (H)­CH spectrum exhibit significantly different intensities for the two signals, indicating that the two conformations of these protons do not occur in a 1:1 ratio. This is in contrast with the previously published data from proton-detected solid-state NMR experiments.^34,35^ Although we cannot confirm the presence of two distinct conformations for the entire TM domain, the histidine side chains closely align with published chemical shifts by L. Andreas and co-workers.^34^ This leads us to the conclusion that these histidine signals correspond to the Nε–Hε atoms of unprotonated H37 of the tetrameric construct, forming hydrogen bonds with the histidines of the adjacent strand, thus resulting in two different signals (Figure 4A, panel 1). Further evidence for this assignment comes from chemical shift predictions based on QM/MM calculations described in the following section. Additionally, the intensity of the Cδ−Hε cross-peak is markedly higher than that of the Cε–Hε cross-peak (Figure S2), further supporting the proposed assignment of Hε.

**Figure 3:**
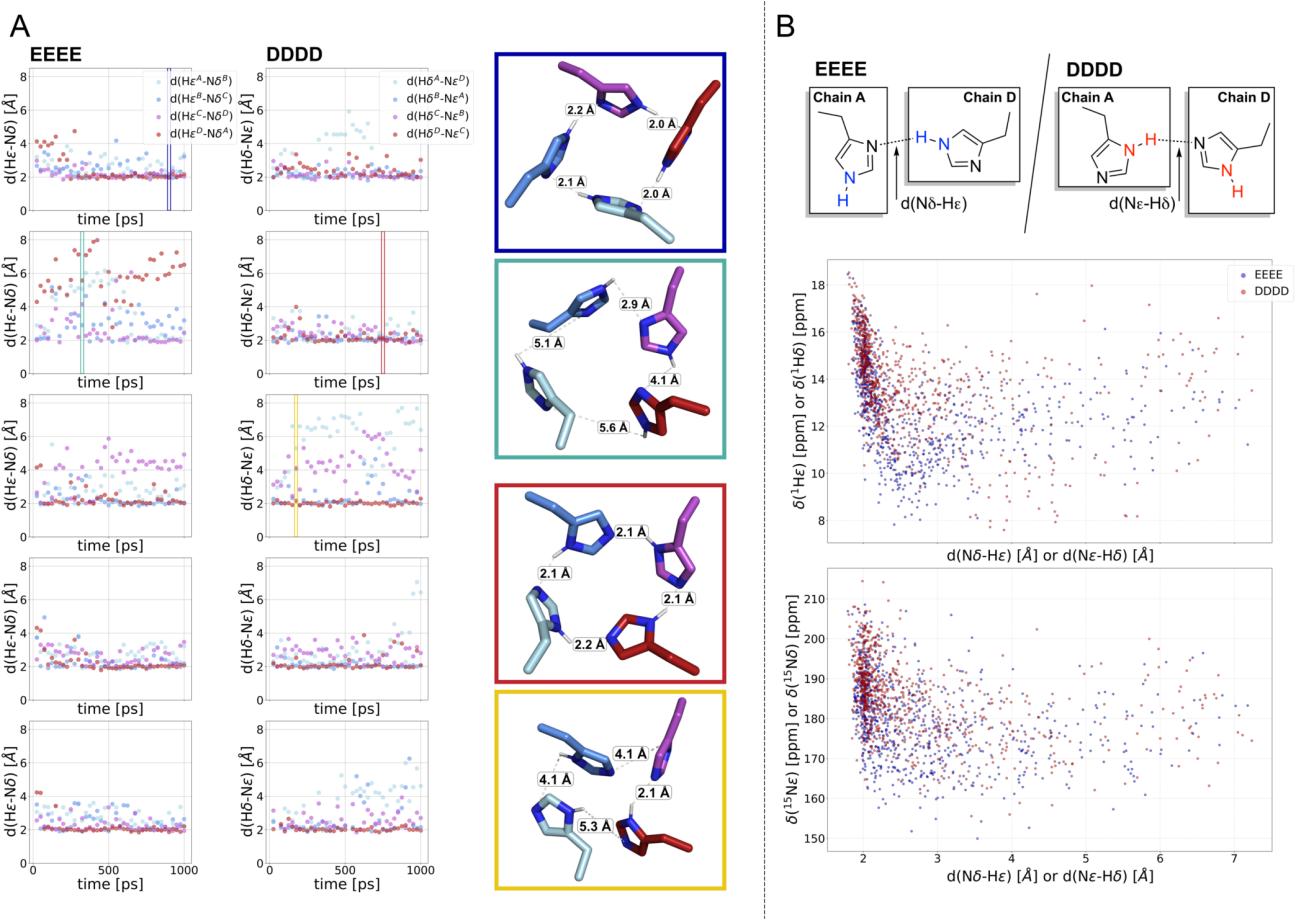
Prediction of the amide proton and nitrogen chemical shifts using QM/MM and cDFT. (A, left panel) Evolution of hydrogen bond distances over time across five replicas of the DFTB2+D simulations. (A, right panel) Representative structures of symmetric and distorted H37 tetrads, corresponding to snapshots indicated in the distance plots.(B, top) Exemplary depiction of hydrogen bonds between Hε-Nδ (EEEE) and Hδ–Nε (DDDD), respectively, along with the definition of hydrogen bond distances (shown explicitly for chains A, D) and relevant nuclei for NMR calculation (highlighted in blue for EEEE and red for DDDD models). (B, bottom panel) H37 NMR chemical shift plotted against the shortest hydrogen bond distance in EEEE (d­(Hε–Nδ)) and DDDD (d­(Hδ–Nε)); chemical shifts in the first graph show ^1^Hε or ^1^Hδ and in the second graph ^15^Nε or ^15^Nδ.

**Figure 4:**
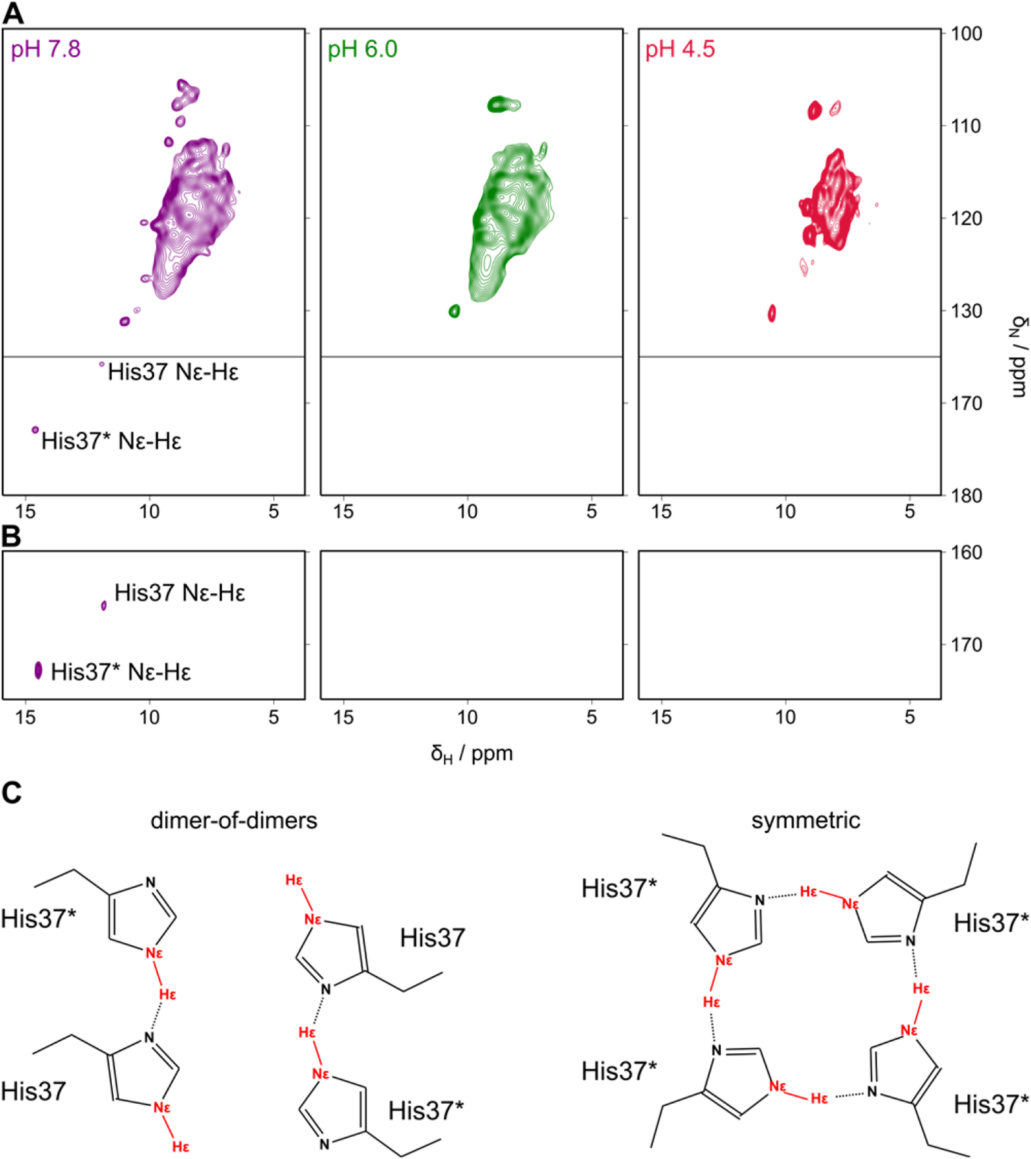
Effects of different pH values on M2. (A) (H)­NH fingerprint spectra based on cross-polarization of the conduction domain of M2 reconstituted into proteoliposomes and measured in buffers at pH 7.8 (purple), pH 6.0 (green), and pH 4.5 (red) with the backbone region (top panel) and the histidine side chain region (bottom panel) shown. For quantitative analysis of chemical shift changes, see Figure 2. (B) Histidine side chain region of INEPT-based (H)­NH spectra for the same samples as shown in (A), highlighting pH-dependent spectral changes. (C) Proposed ideal histidine conformations with hydrogen bonds, depicted in a dimer-of-dimers arrangement (left panel) and a symmetric arrangement (right panel).

The differing intensities at the high pH suggest that the histidine conformations, i.e., hydrogen-bonded and free, deviate from the predicted 1:1 ratio in a hydrogen-bonded, “locked” conductance conformation. Consequently, these differently strong peaks may be caused by binding patterns or side chain dynamics differing from the two ideal states depicted in Figure 4C. One such scenario could be three histidines being involved in hydrogen bond formation, while the fourth is free and experiences different dynamics. Additionally, as previously mentioned, populations of M2 with different protonation states of the histidine tetrad, i.e., a + 1-protonation state, could contribute to the observed ratio as well.

To further investigate the conformation of the histidine tetrad, we recorded three two-dimensional (2D) proton-detected (H)­NH experiments to measure the conformational exchange between the free and hydrogen-bonded states of the H37 side chains in the same sample (Figure S3). We chose exchange times of 0, 2, and 10 s to detect potential interstate exchange and to evaluate the lifetime of ^15^N longitudinal magnetization, which gives us insight into the lifetime of the two histidine protonation states. These experiments showed no cross-peaks, indicating that the tetrad organization of the histidine structure remains stable up to the time scale of tens of seconds. During the exchange time, ^15^N longitudinal magnetization decays due to relaxation and possibly deprotonation and hydrogen exchange. Hydrogen bonds would decrease the HN dipolar coupling of directly bound ^1^H and ^15^N nuclei and limit the motion of the HN bond, which would theoretically decrease the relaxation rate. In our experiments, the signals exhibited an opposite behavior; After 10 s, the signal intensity decreased by approximately 28% for the hydrogen-bonded state and only 12% for the free state. This may be explained by motions of the hydrogen atom between the hydrogen bond donor, i.e., the Nε of H37, and the acceptor, i.e., Nδ of the adjacent histidine.

To evaluate Hε–Nε distance differences between H37 in its free and bound state we then measured one bond ^1^H–^15^N heteronuclear dipolar couplings by cross-polarization (CP) experiments.^55^ The experimental data obtained (Figure S4) was fitted along three variables: dipolar coupling, signal amplitude, and exponential decay, using simulations generated with the SIMPSON software package (version 4.2.1 ^56^). The obtained distances for the bound and free state of H37 are 1.066 and 1.032 Å, respectively, indicating in case of identical local motion a distance elongation by 0.03 Å due to hydrogen bonding. However, the free state is expected to be more flexible, which would result in a stronger reduction of dipolar couplings and a bond difference of less than 0.03 Å. Due to radiofrequency field (RF) inhomogeneities, this technique has a relatively large systematic error, affecting the resulting absolute value. However, the arguably more important result is the difference between the two H–N distances of H37, which because of the equal contribution of this systematic error for both values is less affected.

### Chemical Shift Calculations by QM/MM and cDFT

With our findings from NMR studies, we have a well-founded idea about the histidine tetrad arrangement at pH 7.8. Nevertheless, we are not able to unambiguously assign the NMR signals to a specific side chain conformer or reason whether these side chains are protonated at the Nδ or Nε position. Moreover, we can only deduce the number of hydrogen-bonded and free histidines from the intensity ratio of the respective signals, which apart from concentration in the sample can be influenced by multiple additional factors.

To further investigate the dependency of chemical shifts on the structural properties of H37 and to shed light on the dynamic nature of its protonation, we performed QM/MM simulations of both the EEEE (where all four Nε are singly protonated) and DDDD (where all four Nδ are singly protonated) setups of the tetrad, using a solid-state NMR structure (PDB ID: 2L0J) as the starting configuration. Subsequently, chemical shifts were calculated from snapshots of these simulations at the cDFT meta-GGA level of theory (for details, see Method section). To the best of our knowledge, no combined QM/MM and DFT calculations for chemical shift predictions of the M2 channel have been reported so far despite its frequent use as a model system in NMR-based and computational studies.

The QM/MM simulations of both the EEEE and DDDD configurations revealed a histidine tetrad characterized by strong hydrogen bonds between amide protons and neighboring nitrogen atoms (Figure 3A). However, a symmetry-broken, hydrogen-bond-distorted tetrad pattern with weakened hydrogen bonds was also observed (full trajectory data are shown in Figure S5). These results suggest a dynamic equilibrium in the interactions among the histidines within the tetrad, rather than fully rigid hydrogen bonding, which generally aligns with the conclusion from our solid-state measurements.

To further investigate these interactions, we plotted the predicted ^1^H and ^15^N chemical shifts against the shortest hydrogen bond distances: Nδ-Hε in EEEE and Nε–Hδ in DDDD (Figure 3B). Notably, the deshielding of δ­(^1^Hε) and δ­(^1^Hδ) exhibits a clear trend with decreasing hydrogen bond distance, whereas a reversed trend is observed for Nε–Hε, and Nδ–Hδ distances (Figure S6, Table S2, and S3). When separating the hydrogen shifts based on the hydrogen bond distance, we observed a shift difference of approximately 2 ppm between strongly hydrogen-bonded and non-hydrogen-bonded hydrogen atoms in both the EEEE and DDDD models. This difference closely aligns with the experimental chemical shift difference of 2.68 ppm for the two hydrogens of the H37 side chain, as shown in Figure 3C. Similarly, for the respective nitrogen atoms, the experimental shift difference (Δδ 7.2 ppm) is reasonably reproduced within the error margins, yielding 9.1 and 11.3 ppm for EEEE and DDDD, respectively. These findings support the assumption that hydrogen atoms involved in hydrogen bonding exhibit deshielding of the chemical shifts, in an order of magnitude matching our experimental results. Likewise, the hydrogen-bonded nitrogen atoms experience deshielding, which is associated with a slight elongation of the bond length. In contrast, the non-hydrogen-bonded nitrogen atoms exhibit the opposite trend (Figure S7, Tables S4 and S5). This result aligns well with the experimental CP data, showing a similar trend of bond length difference between hydrogen-bonded and nonbonded NH bonds.

We note in passing that the hydrogen bonding pattern observed in the QM/MM simulations is also in good agreement with the experimentally measured NMR intensities. Both models suggest, on average, more than two hydrogen bonds– exceeding the number expected for the dimer-of-dimers model – which aligns with the stronger signal observed for the hydrogen-bonded histidine (see Figure S8 and Method section for details).

### Structural Changes at Lower pH Values

As M2 is known to be pH-activated, we wanted to investigate its behavior under lower pH conditions, in its active/conducting state. Other studies of the p*K*_a_ value of M2′s histidine side chain, both computational^41^ and experimental,^37,53^ have shown that the active conformation occurs if at least 3 of the imidazole rings of H37 are protonated at a pH ≤ 6.

Using a pH-dependent activity assay, we could show that our construct of M2 is indeed, as formerly described, able to conduct protons in liposomes from lipids and becomes active at a pH < 7. This activation is seen as a decrease in fluorescence as the lumen of the liposome is acidified. As can be seen in Figure S9, the M2-containing sample has the highest rate of proton transport at pH 4, and a similar rate of transport is observed until around pH 6.0. This is in line with the measured p*K*_a_s of 7.0 and 5.5 from Paschke et al.^54^ Decreasing the pH from 7 leads to a minor population of active M2 as the first p*K*_a_ is approached, which corresponds to the minor activity shown at pH 6.5 for the M2 sample. As more M2 tetrads are protonated, maximal activity is reached, as observed from pH 6.0 on. This shows that the setup reported here, both for M2(18–60) and the bilayer, is representative of the wild-type functionality *in vivo*, and that the data at pH 6.0 can be interpreted as a “transitionally active” state.

When reconstituting the channel in this pH 6.0 environment, we discovered that the spectral quality of the solid-state NMR spectra drops, most likely due to the increased structural heterogeneity of the sample (Figure 4A, green spectrum). This hypothesis was supported when analyzing three-dimensional (3D) experiments, which showed broader signals as well as shifted peaks, leading to some residues being unassigned, or at least not clearly identifiable, in the pH 6.0 spectra (Figure S10 and Table S1). ^1^H–^15^N-^13^Cα chemical shift perturbations (CSPs) indicated structural changes in residues close to G34, H37, and of the AH, starting from R45, which became undetectable (Figures S10 and S11).

Aside from the overall spectral broadening and peak pattern changes, the most striking observation was the disappearance of the histidine side chain signals, both in the dipolar coupling-based CP and in the scalar coupling-based INEPT (H)­NH spectrum (Figure 4, second panel). The hydrogen bonds formed at higher pH values seem to no longer be detectable at the lower pH of 6.0. If the protonation state of the histidine tetrad changes from +2 to +3 around pH 6.0, this could further contribute to structural heterogeneity and the observed side-chain dynamics. In addition, the existence of different tautomeric forms or rotamer variations of protonated and deprotonated histidines may promote diverse interaction patterns, thereby enhancing structural inhomogeneity. A previous study by our group in collaboration with Paschke et al.^54^ showed a large tilt of the M2 helix with respect to the membrane between pHs 7 and 6.5. Such large reorientations could contribute to the inhomogeneous structure observed in our spectra at pH 6.0. Moreover, no symmetric hydrogen bond pattern can be formed at a histidine tetrad state of +3. Consequently, the breaking and forming of potential hydrogen bonding patterns could be on a time scale where detection is no longer possible. Interestingly, our results contrast with a study where the histidine side chain peaks were still detected at pH 6.5.^35^ Reasons for this discrepancy could be a variation in measured pH value or different dynamics due to membrane compositions.

Given that the structure of M2 in pH 6.0 is prone to asymmetry and heterogeneity due to its protonation state, we aimed to investigate the channel under a higher protonation state of +3 or +4,^37^ where a symmetric and stable conformation—particularly for the histidine tetrad—is theoretically more likely given that the channel is in its open and fully conducting state.^54^ For that, we measured the NMR spectra of M2 at an even lower pH of 4.5. Interestingly, the spectra showed a more defined structure again, with high-resolution peaks (Figures 2A and 4A, red spectrum) in 2D and 3D experiments. As expected, residue assignments of the channel revealed large chemical shift changes relative to pH 7.8, especially for residues close to G34 and to the C-terminus of the M2 molecule, similar to its resonances at pH 6.0 (Figure S11). Spectral signals for the AH, starting at R45, were no longer detected. The CSP plot (Figure 2B) illustrates these changes, showing large alterations close to G34, at H37 and at the C-terminus of M2 including the AH. Additionally, peak doublings observed at pH 7.8 are not found at pH 4.5. This indicates that the acid-activated conformation of M2 is again more symmetric.

Upon analysis of the backbone dihedral angles, the differences observed between the two conformational states at pH 7.8 and 4.5 were found to lie within the uncertainty margins of the TALOS predictions (Table S6). This suggests that the overall α-helical secondary structure of the transmembrane region of M2 remains largely preserved across the two pH conditions. However, it is important to note that certain structural rearrangements previously reported by some of us at pH 4.5 - based on surface-enhanced infrared absorption (SEIRA) spectroscopy - may not be detectable by MAS solid-state NMR. Therefore, while our current NMR data does not indicate major secondary structural disruptions, we cannot rule out the presence of more subtle or localized conformational changes that may still occur under acidic conditions.

Similar to the sample at pH 6.0, side chain peaks are not observed in HN-correlation spectra for H37 at pH 4.5 and pH 6, however, they are present in the higher pH 7.8 sample (Figure 4A). This implies that the histidine dynamics and conformation expected as part of proton conductance at pH 4.5 are present, at least partially, at pH 6.0. Considering the different protonation states between pH 6.0 and 7.8, it appears the histidines do not form a stable tetrad structure at pH 6.0 and are somewhat dynamic, but not fully conductive. This may indicate a transition state from the “locked” arrangement at pH 7.8 to the ‘conducting’ arrangement seen at pH 4.5.

### MD Simulations Support Channel Opening Under Low pH

Finally, we employed atomistic MD simulations on the microsecond time scale to probe the degree of channel opening and structural changes indicated by the solid-state experiments. Although a number of atomistic MD studies have previously explored the pH-dependent channel opening process, most of the studies were limited to the TM region of M2. A recent study by Torabifard et al.^46^ using constant pH simulations demonstrated, however, that the C-terminal AH plays an important role in pH-dependent conformational changes. To ensure consistency with our solid-state NMR investigations, we performed here classical MD simulations using the same M2 construct as in the experiments including the AH.

To this end, we systematically varied the protonation states of H37. Additionally, D44 of all monomers was protonated at low pH (PPPP*, PPPE*; “*” refers to D44 protonation and “P” to doubly protonated H37) and deprotonated at higher pH. While the protonation state of H37 was a major focus of the current solid-state NMR study, the latter represents a second titratable residue site that was previously shown to influence the p*K*_a_ values of M2.^54^

First, we analyzed the pH-dependent opening and closing dynamics of M2 by calculating the Cα distances between neighboring chains (Figure 5A,B). Compared to the reference solid-state NMR structure (PDB: 2L0J), the results show a clear opening trend at H37 and D44 in simulations of the fully protonated PPPP* or singly deprotonated PPPE* states (Figure 5A). In contrast, simulations of the doubly deprotonated or fully deprotonated states sampled a degree of opening at H37 comparable to the reference NMR structure, while the distance between neighboring D44 decreased. This tightening of the pore upon deprotonation of H37 is further reflected in an overall channel closing, as observed by a reduction in Cα distances at the center (H37), the N-terminus (V27), and the C-terminus (D44) (Table S7) compared with the 2L0J structure.

**Figure 5:**
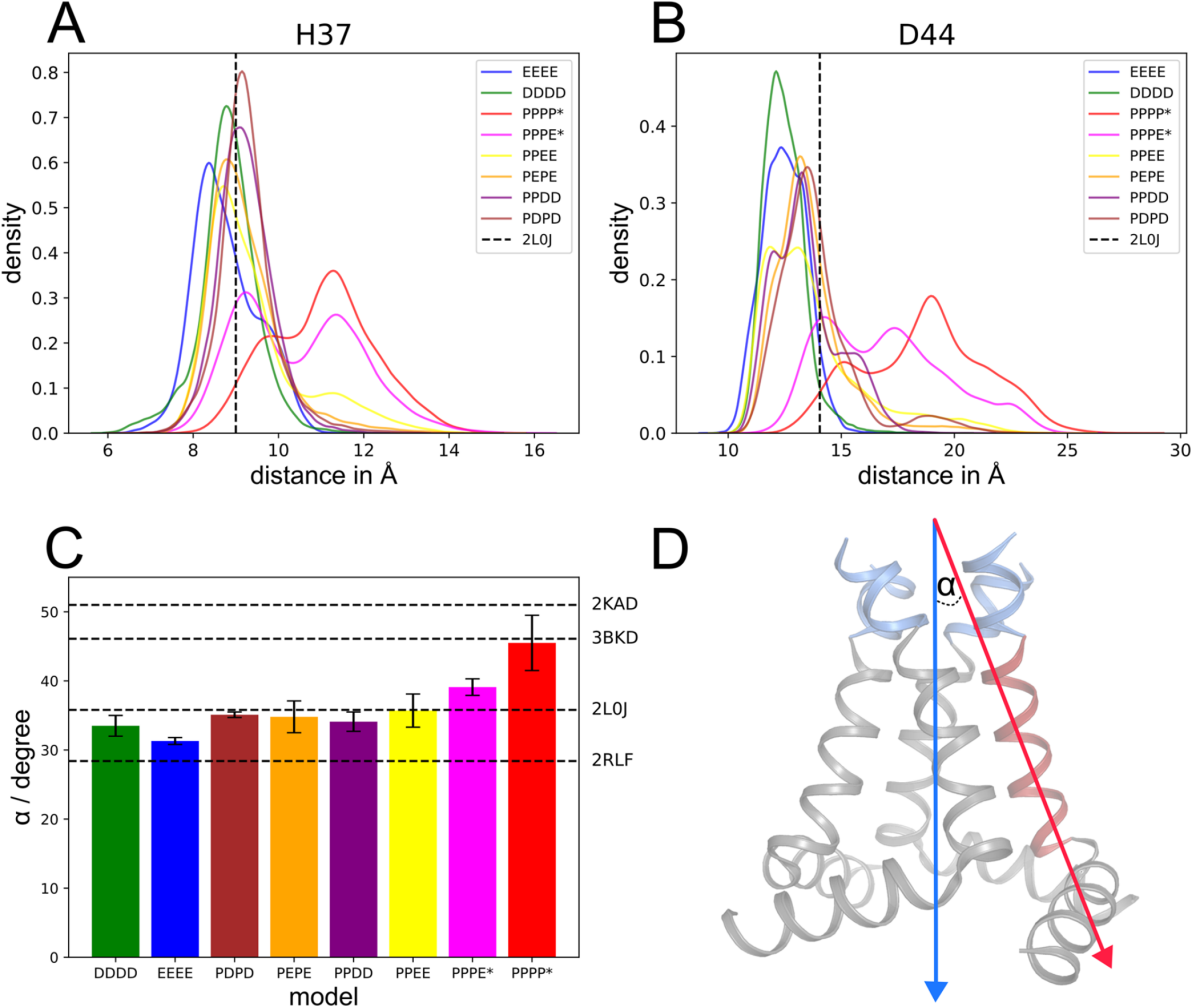
Opening and closing dynamics of M2 observed in atomistic MD simulations. Distance distributions between neighboring monomers measured for (A) H37 and (B) D44 taking the Cα atoms as a reference. The vertical dashed lines indicate the corresponding distances in the solid-state NMR structure (PDB: 2L0J). (C) Average tilt angle between the axis defined by the N-termini (residues 25–32, blue arrow) of all four monomers and the C-termini (residues 33–46, red arrow exemplary shown for one monomer) of the individual chains, as illustrated in (D). Averages were taken over the last 50 ns of all three replicas per model. For comparison, tilt angles from different structures are shown as black dashed lines. Notably, only structure 2L0J (PDB ID) includes the AH, while 2KAD, 3BKD, and 2RLF resolve only the TM domain.

For D44, the distance distributions of the intermediate models (PDPD, PEPE, PPDD, and PPEE) were broader compared to those in the DDDD and EEEE simulations (Figure 5B), indicating increased flexibility of the C-terminal region of the TM helix around D44. This observation aligns with our solid-state NMR data. However, upon protonation of D44 (PPPE* and PPPP*), the stabilizing salt bridge between D44 and R45 of the neighboring monomer was disrupted,^58^ inducing a large change in the tilt angle between the relatively stable N-termini and the C-termini of individual M2 monomers (Figure 5C). This leads to a clear opening of the pore at the C-terminus. The helix orientation observed in the MD simulations is in agreement with the large chemical shift changes seen in our ssNMR data for the C-terminus at pH 7.8 and 4.5, as well as our previous SEIRA measurements on M2.^54^ Comparison of our simulations with known 3D structures suggests that simulations with doubly or fully deprotonated H37 sample an intermediate state similar to the NMR structure (Figure 5C), while simulations of fully protonated PPPP* or singly deprotonated PPPE* states can reach an open conformation comparable to the open-state X-ray structure lacking the AH helix (PDB: 3BKD). This finding is further supported by principal component analysis (PCA) (Figure S12) and is in line with previous computational^41,45,46^ and experimental^37,53^ studies, indicating that the active conformation occurs when at least three H37 are protonated.

## Conclusions

Under the experimental conditions employed—M2-containing proteoliposomes formed from lipids—we confirmed that the conduction domain of M2 forms a nonconducting, homotetrameric structure at pH 7.8. Minor structural disruptions were observed, predominantly localized around transmembrane G34 and C-terminal W41, the latter being implicated in facilitating directional proton transport through the channel. Compared to former studies based on solid-state NMR,^22,31,59^ our data aligns well with the majority of the published results. We interpret the local peak doublings in the N-terminal region of the membrane-spanning helix as slight disruptions of the homotetrameric fold, possibly related to the adjacent unstructured N-terminus and membrane surface.^29^ Peak doublings were also recorded in a previously published study from our group,^54^ and were observed to be more pronounced and appear for more residues than shown here, although not through the whole construct. However, this study was performed in DPhPC bilayers, and only utilized carbon-detected solid-state NMR. This contrast suggests that the lipid environment, either directly through lipid interactions, more generally through membrane viscosity, lateral pressure or hydrophobic thickness, or even through altered local pH conditions, does play an important role regarding subtle structural changes in the M2 channel.^26,30^

Going to acidic pH values, the proton channel very clearly shows large structural changes, leading to a defined conformation of the TM domain not previously described in NMR studies. The heterogeneity at pH 7.8, i.e., doubled resonances, is no longer present. Additionally, the AH seems to either change from the previously recorded defined structure observed in higher pH to a more disordered one or undergo significant dynamic changes, or both in combination, as it is no longer detected at acidic pH. Whereas the large CSPs starting from residue L40 could be directly connected to the altered behavior of the adjacent AH, the larger chemical shift perturbation around G34 is more likely linked to higher structural flexibility in this region, making it very sensible to changes in the environment. Similar observations have been made in previous studies on a transmembrane construct of M2, analyzing the channel in different membrane environments and pH values.^27^ The structural inhomogeneity at pH 6.0, but defined spectra at pH 4.5 indicate that pH 6.0 leads to a transition from closed and nonconducting, to an open and fully conducting state. The functional assay data and MD simulations in this work support this conclusion, with the proton conductance showing a transition between pH 7 and 5.5, and the large pore opening observed by atomistic MD simulations when at least three H37 are protonated.

Concerning detectable H-bonds, experiments at high pH values corroborate the previously described side chain assignments and locked formation in the histidine tetrad of M2.^34^ Different peak intensities though imply that this structural formation is more dynamic than initially described. QM/MM calculations also suggest that the tetrad arrangement is dynamic leading to variation of the hydrogen-bonding pattern of the histidine side chains. This could result in tetramers where 2,3, or even 4 of the four histidines are connected via H-bonds, depending on external factors such as the lipid environment or local pH variation, and may explain the lack of a 1:1 intensity ratio observed in the NMR spectra.

The channels’ histidine tetrad conformation and dynamics seem to change significantly when they are activated in acidic conditions. Taken together with the disappearance of peak doubling for the protein backbone, this suggests a mechanism of proton conduction where the flexible histidine chains, no longer locked in a rigid arrangement, mediate the conduction of the protons through protonation/deprotonation (Figure 6). This mechanism would be in line with the earlier proposition from Hong, de Grado, Voth, and colleagues.^41,43^

**Figure 6:**
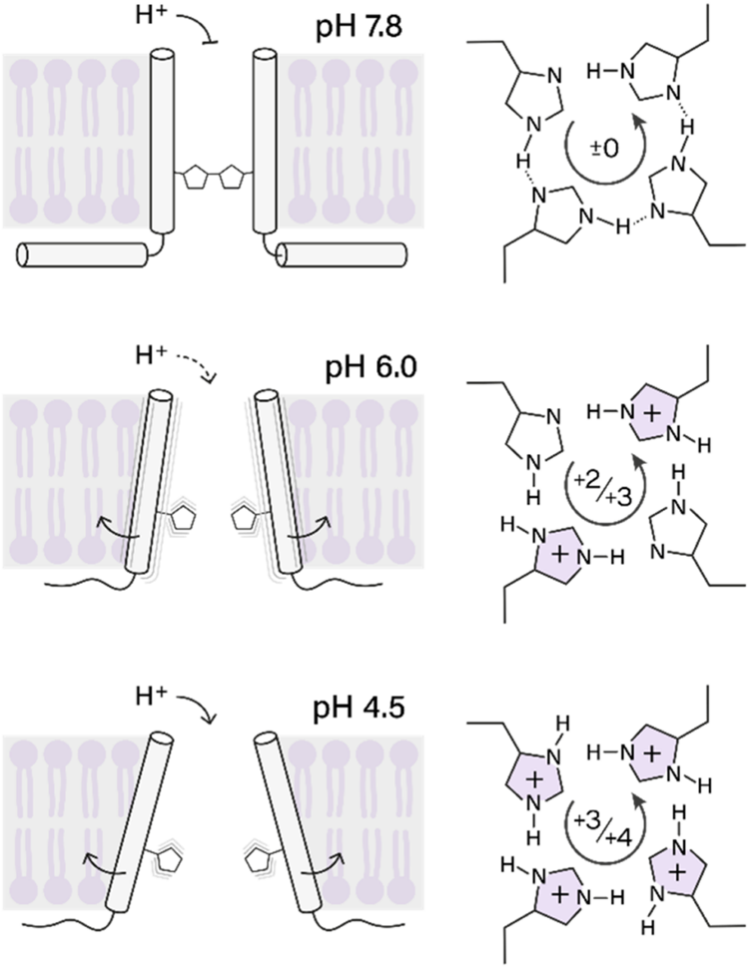
Suggested model of M2 activation. At pH 7.8, the channel adopts a closed, well-ordered conformation with defined transmembrane (TM) and amphipathic helices (AH). Hydrogen bonds are present between His37 side chains, occasionally breaking to give rise to two NMR signals, yet without a major disruption of the homotetrameric structure. At pH 6.0, the channel exhibits increased structural heterogeneity throughout the TM region, likely promoted by the shift from a + 2 to a + 3 protonation state of the histidine tetrad. The AH shows increased dynamics and a loss of helical definition. No hydrogen bonding between histidines is observed. At pH 4.5, the TM domain regains a more defined structure, while the AH and histidine side chains retain characteristics similar to those at pH 6.0. The schematic representations of tilt angle changes and pore expansion are based on our previously published data^54^ and are included here to provide a conceptual summary.

The unexpected high resolution observed here for the first time for proton-detected spectra of M2 at pH 4.5 will allow future investigations into the open structure, including distance measurements and potentially structure calculations.

## Experimental Methods

### Protein Expression

The IAV M2 18–60 construct (i.e., M2CD, in the following simply referred to as “M2”) was expressed in inclusion bodies as a C-terminal fusion with the (His)­9-trpLE polypeptide by overexpression in Bl21­(DE3) as previously described by Chou and others.^54,60^ A D_2_O-adaptation protocol following the method published by Fricke et al.^61^ was used to achieve complete deuteration of the protein: A single colony of freshly transformed Bl21­(DE3) cells was used to inoculate a mixture of 5 mL (protonated) LB medium with 5 mL (deuterated) M9 medium. After incubation at 37 °C for 2–3 h, this culture was diluted with 10 mL of deuterated M9 medium and incubated for a further 2–3 h. The complete culture was then further diluted with 280 mL of deuterated M9 medium and incubated at 30 °C for 16 h. Bacteria were spun down at 3000 rcf for 10 min and used to inoculate 2 L of fresh deuterated M9 medium. This culture was incubated at 37 °C to an OD_600_ of 0.6–0.7 and the temperature was lowered to 18 °C followed by induction by the addition of 0.15 mM IPTG. Expression was carried out at 18 °C for at least 16 h. Bacteria were collected by centrifugation (5000 rcf, 30 min) and stored at −80 °C. Protein purification and cyanogen bromide (CNBr) cleavage were performed as published.^54,60^

Similarly, to produce deuterated lipids, untransformed Bl21­(DE3) were adapted to fully deuterated M9 medium by stepwise dilution and media exchange. Bacteria were incubated to the stationary phase, harvested by centrifugation (5000 rcf for 30 min), and lyophilized to complete dryness. Lyophilized bacterial pellets were dissolved in a monophasic mixture of chloroform: methanol: H_2_O (1:1:0.8 vol/vol/vol) and incubated for 1 h with vigorous stirring. By the addition of appropriate amounts of chloroform and H_2_O, the mixture was brought to a two-phase state.^62^ The lower (chloroform) phase was isolated, dried, and further purified by another round of monophasic-to-biphasic transition and chloroform phase isolation. The purified deuterated lipids were dried and resuspended in ultrapure H_2_O, lyophilized, and stored at −20 °C under an argon atmosphere.

Purified, lyophilized M2 and deuterated lipids (1:1 w:w ratio) were added to denaturing buffer (6 M guanidine, 40 mM phosphate, 30 mM glutamate, 3 mM sodium azide, pH 4.5, pH 6.0 or pH 7.8, ≥33 mg/mL OG detergent) and dialyzed against 1 L sample buffer (40 mM phosphate, 30 mM glutamate, 3 mM sodium azide, pH 4.5, pH 6.0 or pH 7.8) for 7 days with 2 dialysis buffer changes per day.

### Activity Assay

The activity assay described here is adapted from Pielak et al.,^63^ with modifications to enable a fluorescence measurement. Purified, lyophilized M2 was resuspended in organic solvent equivalent to the high-performance liquid chromatography (HPLC) elution conditions (38.7:26.4:36.9 (v:v:v) Acetonitrile:Isopropylalcohol:H_2_O) to a 1 mg/mL solution. This was stored under inert gas (N_2_) at −20 °C for at most 2 months prior to use. To prepare liposomes, 2 mg total extract lipids (from a 25 mg/mL chloroform stock purchased from Avanti Polar Lipids, Alabaster, AL) were added to a glass vial along with 100 μg (400 nmol) M2 (for the M2-containing sample) and 8 nmol valinomycin (from a 50 μM stock in ethanol purchased from Cayman chemicals). The resulting organic mixture was dried under N_2_ stream, and kept under high vacuum for at least 12 h, and stored in inert conditions (N_2_) at −20 °C for at most 1 week prior to use.

Lipid film (LUVs: lipid and valinomycin, M2: M2, lipids, and valinomycin) was resuspended in 100 μM pyranine, 50 mM sodium phosphate, 50 mM citrate, 122 mM KCl, and 122 mM NaCl, pH 7.7, and incubated at room temperature overnight, with agitation. Lipid mixtures were then subjected to 5× freeze–thaw cycles, with the thaw cycle at 37 °C, and extruded at least 31× through a 400 nm filter to produce homogeneous 400 nm LUVs. Liposomes were then buffer exchanged to 5 mM sodium phosphate, 5 mM citrate, 122 mM KCl, and 122 mM NaCl, pH 7.7, and diluted to a final lipid concentration of 133 μg/mL and used immediately.

Fluorimetry measurements were performed on a JASCO FP-6500 fluorimeter, with an excitation at 450 nm and emission recorded at 510 nm every 1 s, temperature was controlled by water bath to 37 °C. One mL portion of the sample was used per measurement, with a stirrer, and performed in technical triplicate. Baseline initial fluorescence was recorded for 30 s, prior to injection of 500 μL of “Initiation buffer” (50 mM sodium phosphate, 50 mM citrate, 122 mM KCl, and 122 mM NaCl, pH set to titrated pH desired). Decrease in fluorescence was then recorded until the addition of 500 μL of “End buffer” (10% Triton, 50 mM sodium phosphate, 50 mM citrate, 122 mM KCl, 122 mM NaCl, pH set to desired end pH) at 160 s. The end stable state was recorded for 240 s. Calculation of rate was calculated by linear regression of 20 s time window, from injection of “Initiation buffer” as observed as an initial sharp decrease in fluorescence due to dilution.

### NMR-Experiments and Analysis

For proton-detected experiments, the fully ^13^C,^15^N-labeled, deuterated, and then back-exchanged M2 reconstituted in a pH 7.8, 6.0, or 4.5 phosphate buffer was packed into 1.3 mm rotors. Measurements were performed on a wide-bore 600 MHz Bruker NMR spectrometer equipped with a 1.3 mm 3-channel probe (Bruker BioSpin) and on a standard-bore 900 MHz Bruker NMR spectrometer equipped with a 1.3 mm 4-channel VTX probe (Bruker BioSpin). Measurements were performed under 55 kHz magic angle spinning. For H–N exchange spectra and ^1^H–^15^N dipolar coupling measurements, the sample at pH 7.8 was packed into a 1.9 mm rotor and measured under 40 kHz spinning on a 700 MHz Bruker NMR spectrometer equipped with a 1.9 mm four-channel probe (Bruker BioSpin). The deuterium signal was used for the external field-frequency lock.

The 3D assignment experiments ((H)­CαNH, (H)­CONH, (H)­Cα­(CO)­NH, and (H)­CO­(Cα)­NH) were performed according to the previously published procedure^61^ (Figure S1). Instead of using CP and DREAM transfers for C–N and C–C correlations, optimized control pulses were used.^64,65^ For the H–N exchange experiments, the pulse sequence used can be found in the SI (Figure S3). The pulse sequence for ^1^H–^15^N dipolar coupling measurements can be found in the SI (Figure S4). A contact time of the first CP period was incremented in 50 μs steps up to 1.0 ms to monitor the ^15^N magnetization dynamics driven by the recoupled dipolar interaction. During these CP steps, nitrogen and proton RF fields were applied with a constant amplitude of approximately 30.5 and 10.5 kHz, respectively. Peak volumes of amide resonances in 2D spectra were calculated by a manual box integration routine implemented in TOPSPIN 4.1.1. (Bruker BioSpin). The software package SIMPSON 4.2.1 ^56^ was used to provide best-fit simulations using an in-built multiparameter minimization protocol.

All experiments took place at a sample temperature of approximately 20 °C, which was determined using the water ^1^H resonance chemical shift and DSS (4,4-dimethyl-4-silapentane-1-sulfonic sodium salt) as an internal chemical shift.^66^ For pH 6.0, no C chemical shifts were assigned due to poor spectral quality. The N, Cα and H assignments were based on previous data from the pH 4.5 sample, and results from previously published work.^54^

For the proton detection experiments, either a MISSISSIPPI water suppression^67^ or a water suppression using a spoil pulse followed by a train of saturation pulses^61^ was used. Acquisition and processing as well as data analysis were done using Topspin 4.1.1 (Bruker BioSpin). Assignments and further analysis were done using CCPNMR 3.2.0.^68^

For the chemical shift analysis regarding protein backbone torsion angles, the TALOS+ software^57^ was used.

For structural visualization, an AlphaFold prediction of the used M2 sequence (residue 18–62) was calculated with the help of the AlphaFold v2.3.2 colab web implementation.^69^

^1^H–^15^N–^13^Cα chemical shift perturbations were calculated as Euclidian distances (eq 1)^70^

1 $$\mathrm{CSP}=\sqrt[{2}]{\frac{1}{3}\left(\delta_{H}^{2}+\alpha \cdot \delta_{N}^{2}+{\beta \cdot \delta }_{\mathrm{Ca}}^{2}\right)}$$

where δ_H_, δ_N_, and δ_Ca_ are the chemical shift differences between M2 at pH 7.8 and at pH 4.5 or 6.0 respectively. α is 0.2 for glycine residues and 0.14 for all other amino acids, β is 0.3.

### Hybrid Quantum Mechanic/Molecular Dynamics Simulations and NMR computations

All DFTB2 and DFTB3 QM/MM simulations were performed using the built-in QM/MM extension^71–73^ in connection with the sander routine of AMBER version 22.^74^ Throughout, the MIO-1–1 ^75^ and 3OB^76^ parameter sets are used for DFTB2 and DFTB3 simulations, respectively. GFN2-xTB^77,78^ QM/MM simulation use the AMBER internal interface with an external QM code^79^ and Orca, program version 6.0.^80,81^ We employ an adapted version of the implemented dispersion model for DFTB2 + D,^82^ allowing more interactions than in the original parametrization to prevent equilibration runs from aborting due to excess contacts; the adapted parameter files are included in the Supporting Information. We note in passing that the direct comparison of production runs with the adapted and original dispersion corrections in EEEE gave the exact same trajectories; we thus used the adapted file throughout. For all simulations, a symmetric protonation of histidines at the Nε position or Nδ position was employed.

All QM/MM runs are based on the PDB: 2L0J structure,^22^ embedded in a DOPC bilayer, water shell, and 0.15 M neutralizing KCl as obtained from the CHARMM-GUI web-interface.^83–85^ The initial structure was optimized for 3000 steps with a restraint weight of 100 kcal · mol^–1^ ·Å^–2^ on the non-QM atoms, followed by unrestrained minimization of 5000 steps. Throughout, the histidine tetrad (H37) excluding the backbone atoms (C_α_, C=O, and N–H) was chosen as the QM region (44 atoms). Equilibration runs were conducted for 100 ps using a 1 fs integration time-step (100,000 steps) within an NPT ensemble to optimize the box size and stabilize the pressure before the final production runs. QM atoms were constrained (100 kcal · mol^–1^ · Å^–2^) during the equilibration runs. Three replicas of the 1 ns production run (1000,000 steps; integration time-step of 1 fs) were performed, from which for each 40 equidistantly separated snapshots were chosen for the subsequent NMR calculations (one snapshot every 25 ps, starting at 25 ps).

The QM region for NMR calculations was built as follows: The QM region of the DFTB2 + D QM/MM production runs was used as the “central” QM atoms. Additionally, all atoms within 3 Å of the central QM atoms were included as the “near-field” QM atoms, and all water molecules and protein residues of which at least one atom is within a 6 Å sphere of the central QM atoms are included in the “far-field” QM atoms. Lipids are included if at least one non-hydrogen atom is within a 6 Å sphere (isolated hydrogens are excluded). To avoid the inclusion of full lipid molecules, the lipid fragments are capped at the next carbon. Here, a few exceptions apply: if any heteroatom is within the selection, these groups are included fully, also double-bonded carbons are included (to avoid terminal sp^2^ hybridized carbons). If two fragments are separated by less than two carbon atoms, i.e., they will share a cap, this cap is also included in the far-field QM atoms. The selection is saturated with all bonded hydrogens and capped, as described above. All atoms within 10 Å spheres around the full QM region are included as a point charge environment represented by point charges extracted from the CHARMM force field. We note in passing that this setup is similar to automated fragmentation QM/MM and (field-adapted) adjustable density matrix assembler-based NMR protocols developed in the past.^85–88^

All QM NMR calculations were performed on TPSS^89^ level of theory in its current-density functional generalization via the Dobson model for the kinetic energy density,^90,91^ as implemented in the Turbomole program code version 7.7.1..^92,93^ Central and near-field QM atoms are treated with the pcSseg-2 basis, far-field QM atoms use pcSseg-1 basis sets,^94^ in all cases “universal” auxiliary basis sets are employed.^95^ The resolution of the identity within its multipole accelerated implementation is used for the Coulomb interactions throughout.^96^ An exemplary representation of the QM region within the PC environment and the central, near, and far-field QM regions are shown in Figure S12.

Throughout, shifts are referenced against TMS (^1^H) or NH_3_ (^15^N). Both reference structures are based on DFTB2 gas-phase structures obtained with the SQM routine of AMBER; optimization of NH_3_ additionally used the implemented dispersion correction, which due to missing parameters was not employed for TMS. For silicon interactions with hydrogen and carbon the PBC-0–3 ^97,98^ parameters were used throughout. Both references were corrected by the gas-to-liquid shifts as tabulated for TMS^99^ and NH_3_ ^100^ (2.032 and 18.15 ppm, respectively), TMS employs an additional correction for the liquid-to-solution shift for 1% solution of TMS in CDCl_3_ (0.665 ppm) as listed in Table 1 of ref 99.

**Table 1:** Overview of All Models

name	H37	D44	atoms	DOPC	DOPE	water	K^+^	Cl^–^	time/ns	replicas
DDDD	Nδ, Nδ, Nδ, Nδ	deprot	71,927	172	43	13,270	35	43	1000	3
EEEE	Nε, Nε, Nε, Nε	deprot	71,951	172	43	13,262	35	43	1000	3
PDPD	Nε,δ, Nδ, Nε,δ, Nδ	deprot	71,916	172	43	13,257	35	45	1000	3
PEPE	Nε,δ, Nε, Nε,δ, Nε	deprot	71,895	172	43	13,250	35	45	1000	3
PPDD	Nε,δ, Nε,δ, Nδ, Nδ	deprot	71,964	172	43	13,273	35	45	1000	3
PPEE	Nε,δ, Nε,δ Nε, Nε	deprot	71,931	172	43	13,262	35	45	1000	3
PPPE*	Nε,δ, Nε,δ, Nε,δ, Nε	prot	71,983	172	43	13,276	35	50	1000	3
PPPP*	Nε,δ, Nε,δ, Nε,δ, Nε,δ	prot	71,928	172	43	13,257	35	51	1000	3

In the Results and Discussion Section, we focused on the interaction dynamics and resulting hydrogen bonding-induced shifts. However, on directly comparing the experimental and computed chemical shifts based on the outlined protocol (Figure S8A), we found that the simulated absolute ^1^H values underestimate experimental shifts for the hydrogen-bonded histidine signal in EEEE but overestimate the noninteracting chemical shifts in DDDD. The agreement between the EEEE prediction and experimental shifts is slightly better than that of the DDDD configuration. To account for the different hydrogen bonding states, we separated distances below and above 2.5 Å, assuming that distances below this threshold correspond to interacting (i.e., hydrogen-bonded) histidines, while those above represent noninteracting histidines (Figure 3C). Calculated ^15^N shifts in all cases are overestimated; however, EEEE reaches the vicinity of the experimental shift scale and hence appears to agree better with experimental results. Nevertheless, the presence of either the protonation state or a tautomeric structure, as suggested in ref 34 cannot be ruled out by these results.

Furthermore, we compared the average number of hydrogen-bonding interactions in the histidine tetrad during the DDDD and EEEE simulations. As shown in Figure S8B, the average number of hydrogen bonds in DDDD is slightly higher than in EEEE. However, in both models, the average exceeds two hydrogen bonds expected for the dimer-of-dimers conformation of the tetrad (Figure 4C, left). In relation to the experimental results, the intensity of the hydrogen-bonded histidine signal appears to be stronger, which aligns with the observed average of more than two hydrogen bonds. Thus, this finding is reasonably well reproduced in both models.

### Molecular Dynamics Simulations

The 3D structure of the M2 homotetramer was extracted from the protein database (PDB: 2L0J, model 1). The model was prepared with the CHARMMGUI web server.^88^ In the first step, the protonation for each model was defined, as indicated in Table 1. Then, the models were inserted in a 90 × 90 Å^2^ DOPC:DOPE (ratio: 4:1) membrane, solvated with TIP3P water molecules,^101^ and neutralized with 150 mM K^+^Cl^–^.

The following MD simulations were performed with Gromacs 2021.2 ^102^ using the Charmm36m force field.^103^ At first, the models were prepared by applying the multistep protocol generated by CHARMMGUI. This includes steps of energy minimization and equilibration with stepwise decreasing position restraints. Subsequently, the production dynamics of 1 μs was run in an NPT ensemble at 300 K and 1 atm. The temperature and pressure were controlled by the velocity rescaling method^104^ and the Berendsen barostat,^105^ respectively. Short-ranged electrostatics and van der Waals interactions were truncated above 1.2 nm. Long-ranged electrostatics were enabled with the Particle mesh Ewald summation^106^ and periodic boundary conditions were applied in all directions. To ensure stability, a time step of 2 fs was used and all bonds containing hydrogens were constraints using LINCS.^107^ For statistics, all simulations were independently repeated three times.

### Post MD Analysis

The conformation of the M2 pore was described by two parameters, namely, the distances between neighboring monomers and a principal component analysis (PCA).

The distances between neighboring monomers were determined by taking the Cα atoms of different positions of the M2 channel into account. Main emphasis was put on the following residues of the M2 pore: (a) the N-terminus (V27), (b) the center (H37), and (c) the C-terminus of the TM helices (D44). This analysis was done with the tcl interface of VMD.^108^

A principal component analysis (PCA) was performed using the Gromacs toolbox. For this, the trajectories of the DDDD and PPPP* models were used to determine the covariance matrix and the eigenvectors describing the most important motions. In this way, we aim to extract the most extreme motions of M2 in the pore opening dynamics. Subsequently, all combined trajectories of all individual models were projected on these motions (eigenvalues) to investigate which states of the opening-closing, the reaction coordinate, are sampled by each individual model.

Besides these two most important descriptors, the tilting of the C-terminal parts of helix TM (33–46) was calculated with respect to the axis defined by the N-terminal region (25–32) of all four TM helices. Moreover, for visualization of the pore size, the distances between the neighboring monomers defined above were summed as the perimeter for V27, H37, and D44.

## Supplementary Material



## Notes

TALOS+ calculation results, additional NMR spectra, additional simulation data, and the results of the activity assay (PDF)

## Abbreviations

LUV: large unilamellar vesicle
DPhPC: 1,2-diphytanoyl-*sn*-glycero-3-phosphatidylcholine
