# Supplementary material for: Structural Transition from Closed to Open for the Influenza A M2 Proton Channel as Observed by Proton-Detected Solid-State NMR
Source: J Am Chem Soc. 2025 Jun 20;147(31):27537–51. doi: 10.1021/jacs.5c05111 (PMC12333377; doi:10.1021/jacs.5c05111)
Supplement: Supplementary file 1 [file ja5c05111_si_001.pdf]

Supporting Information for

Structural transition from closed to open for the Influenza A M2 proton channel as observed by proton-detected solid-state NMR

Swantje Mohr[1], Caspar Schattenberg[2], Tillmann Utesch[2], Henry Sawczyc[1], Veniamin Chevelkov[1], Sascha Lange[1], Jacek Kozuch[3], Han Sun[2,4*], and Adam Lange[1,5*]

[1]Research Unit Molecular Biophysics, Leibniz Forschungsinstitut für Molekulare Pharmakologie (FMP), 13125 Berlin, Germany.

[2]Research Unit Structural Chemistry and Computational Biophysics, Leibniz Forschungsinstitut für Molekulare Pharmakologie (FMP), 13125 Berlin, Germany.

[3]Experimental Molecular Biophysics, Physics Department, Freie Universität Berlin, 14195 Berlin, Germany.

[4]Institut für Chemie, Strukturelle Chemische Biologie und Cheminformatik, Technische Universität Berlin, 10623 Berlin, Germany.

[5]Institut für Biologie, Humboldt-Universität zu Berlin, 10115 Berlin, Germany.

Table S1: Chemical shift assignments for the M2 (18-60) conduction domain, acquired at pH 7.8, 6.0, and 4.5. Double / triple resonances are shown in the "chemical shift*" column.

			pH 7.8		pH 6.0	pH 4.5
Residue Nr.	Residue	Atom	chemical shift / ppm	chemical shift* / ppm	chemical shift / ppm	chemical shift / ppm
24	ASP	H	8.81		9.43	
24	ASP	N	125.44		127.26	
24	ASP	CA	51.78		53.39	
24	ASP	C				
25	PRO	N				
25	PRO	CA				
25	PRO	C	177.27			176.82
26	LEU	H	8.62		8.67	8.73
26	LEU	N	118.24		118.37	118.89
26	LEU	CA	57.75		58.01	57.97
26	LEU	C	178.26			176.50
27	VAL	H	8.08		8.02	8.11
27	VAL	N	120.59		120.57	121.47
27	VAL	CA	66.29		67.14	66.71
27	VAL	C	179.00			177.39
28	VAL	H	8.48		7.81	8.04
28	VAL	N	119.93		119.68	120.24
28	VAL	CA	66.61		66.85	66.91
28	VAL	C	177.85			176.35
29	ALA	H	8.49	8.58	8.43	8.45
29	ALA	N	119.84	120.74	120.56	120.65
29	ALA	CA	55.33	55.48	55.28	55.25
29	ALA	C	178.47	178.40		173.90
30	ALA	H	8.27	8.87	8.66	8.61
30	ALA	N	117.28	117.84	117.85	117.92
30	ALA	CA	55.02	54.63	54.24	54.41
30	ALA	C	178.47	178.32		
31	SER	H	8.15	8.25	8.52	8.68
31	SER	N	112.61	112.60	112.66	111.21
31	SER	CA	62.46	62.08	62.68	61.39
31	SER	C	175.33	175.31		177.98
32	ILE	H	8.50		8.09	8.07
32	ILE	N	120.35		118.26	118.17
32	ILE	CA	62.94		65.45	65.10
32	ILE	C	177.05			176.55
33	ILE	H	7.43		8.60	8.31
33	ILE	N	118.90		119.40	119.09
33	ILE	CA	65.85		64.23	64.23
33	ILE	C	176.99			178.05
34	GLY	H	8.65	8.39 / 8.75	8.88	8.92

34	GLY	N	105.62	106.50 / 109.48	107.81	108.47
34	GLY	CA	48.08	47.41 / 46.52	47.12	47.59
34	GLY	C	175.11			174.45
35	ILE	H	8.17		7.84	7.92
35	ILE	N	119.89		121.02	121.07
35	ILE	CA	64.84		65.36	65.03
35	ILE	C	176.85			176.61
36	LEU	H	8.66		8.60	8.59
36	LEU	N	119.48		119.31	119.53
36	LEU	CA	58.00		57.89	57.79
36	LEU	C	177.66			178.19
37	HIS	H	8.70		8.54	8.37
37	HIS	N	116.56		116.09	115.61
37	HIS	CA	59.23		60.09	60.09
37	HIS	C	175.16			176.77
37	HIS	Nδ/ε	172.91	165.71		
37	HIS	Hδ/ε	14.62	11.94		
37	HIS	Cδ	113.09	114.05		
37	HIS	Cε	131.48	134.13		
38	LEU	H	7.95		8.11	8.10
38	LEU	N	117.39		117.62	117.51
38	LEU	CA	58.53		58.59	58.56
38	LEU	C	177.52			178.26
39	ILE	H	8.23		8.24	8.40
39	ILE	N	114.31		113.98	113.81
39	ILE	CA	64.86		62.51	62.39
39	ILE	C	177.14			
40	LEU	H	9.10		8.26	8.05
40	LEU	N	121.47		122.27	121.67
40	LEU	CA	58.16		58.19	58.17
40	LEU	C	178.69			178.69
41	TRP	H	9.71	8.80	9.22	9.10
41	TRP	N	120.82	123.12	122.52	121.97
41	TRP	CA	60.99	62.29	60.78	60.67
41	TRP	C	178.27			177.13
42	ILE	H	8.04		8.71	8.65
42	ILE	N	117.20		117.47	117.35
42	ILE	CA	66.11		65.64	65.15
42	ILE	C	177.85			176.46
43	LEU	H	8.75		8.39	8.45
43	LEU	N	117.31		119.30	119.33
43	LEU	CA	57.66		57.30	57.38
43	LEU	C	179.68			177.59
44	ASP	H	9.33		9.36	9.198

44	ASP	N	120.58		120.27	118.97
44	ASP	CA	57.48		57.07	56.36
44	ASP	C	178.35			
45	ARG	H	8.74			
45	ARG	N	116.47			
45	ARG	CA	56.68			
45	ARG	C	178.39			
46	LEU	H	8.03			
46	LEU	N	114.26			
46	LEU	CA	56.11			
46	LEU	C	177.45			
47	PHE	H	7.74			
47	PHE	N	112.29			
47	PHE	CA	57.90			
47	PHE	C	175.62			
48	PHE	H	7.45			
48	PHE	N	116.62			
48	PHE	CA	60.64			
48	PHE	C	175.80			
49	LYS	H	10.18			
49	LYS	N	120.49			
49	LYS	CA	56.78			
49	LYS	C	175.59			
50	SER	H	9.29			
50	SER	N	111.89			
50	SER	CA	60.36			
50	SER	C	174.76			
51	ILE	H	9.95			
51	ILE	N	127.88			
51	ILE	CA	64.23			
51	ILE	C	176.44			
52	TYR	H	8.05			
52	TYR	N	117.49			
52	TYR	CA	62.32			
52	TYR	C	178.90			

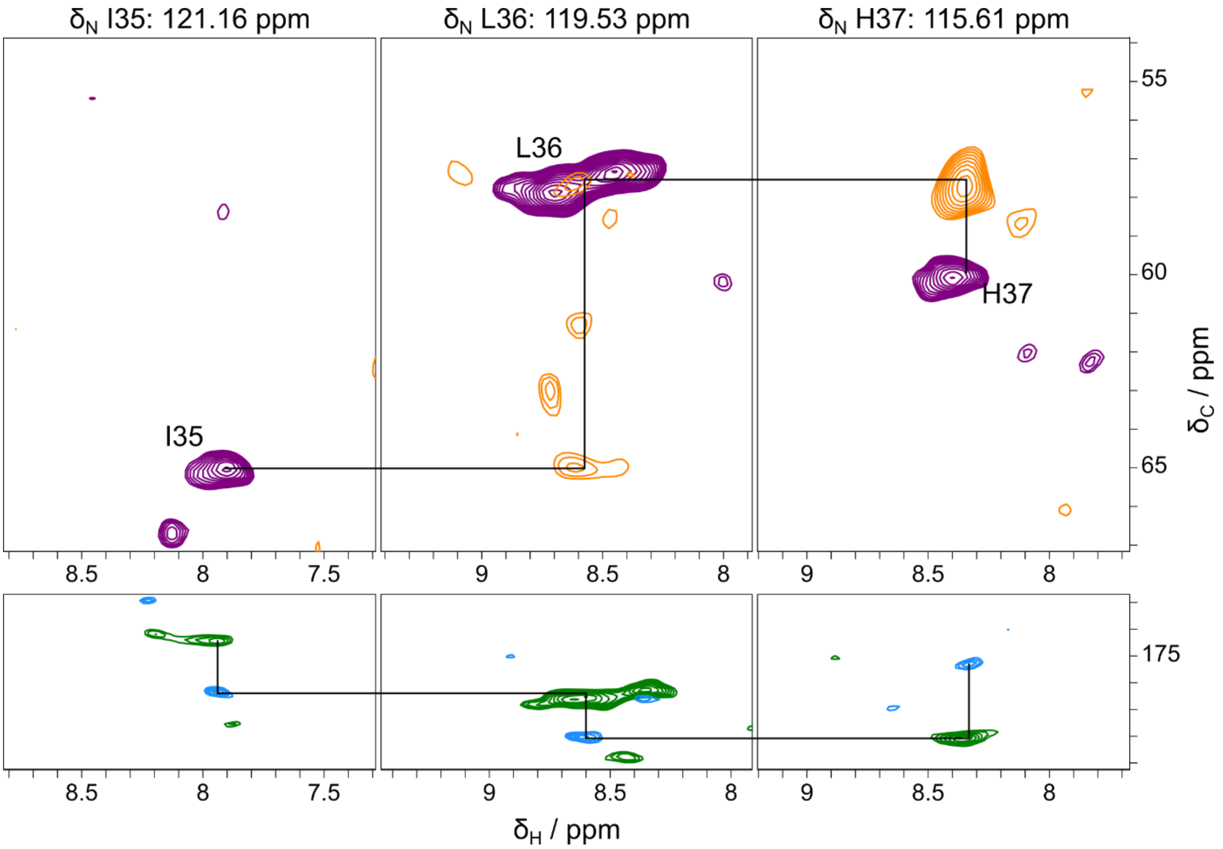

Figure S1: Sequential walk for the amino acid stretch I35 to H37. The (H)CαNH spectrum is shown in purple, the (H)Cα(CO)NH in orange, the (H)CONH in green, and the (H)CO(Cα)NH in blue. Lines represent the connections used in the assignment process.

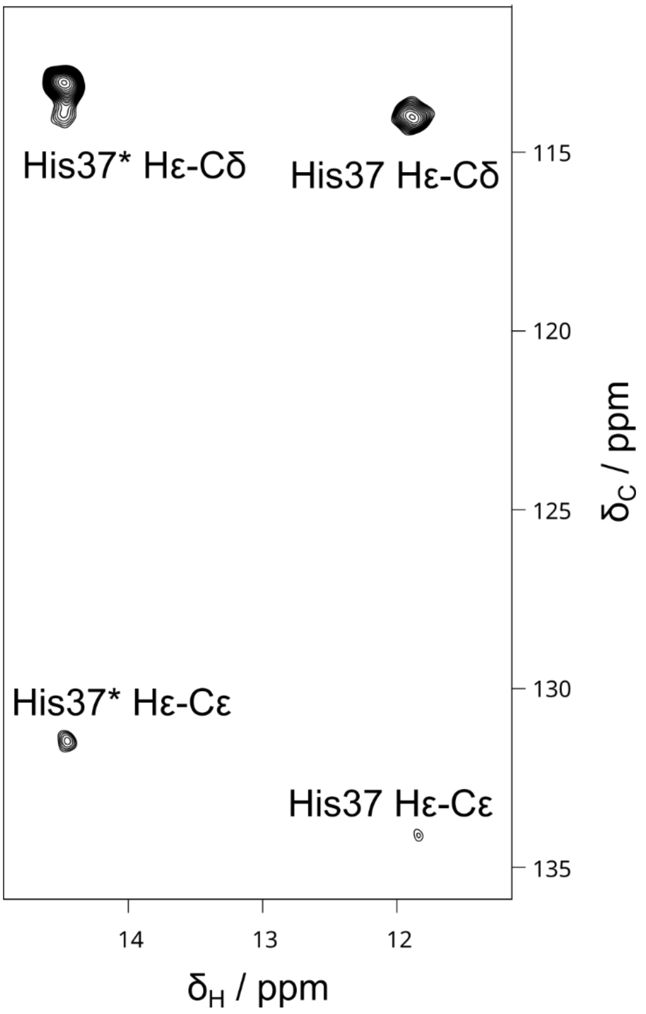

Figure S2: CP based (H)COH spectrum of M2 at pH 7.8. Suggested assignments based on literature values (Li & Hong, main text ref. 53) of the recorded cross-peaks are marked according to the nomenclature used in Figure 4.

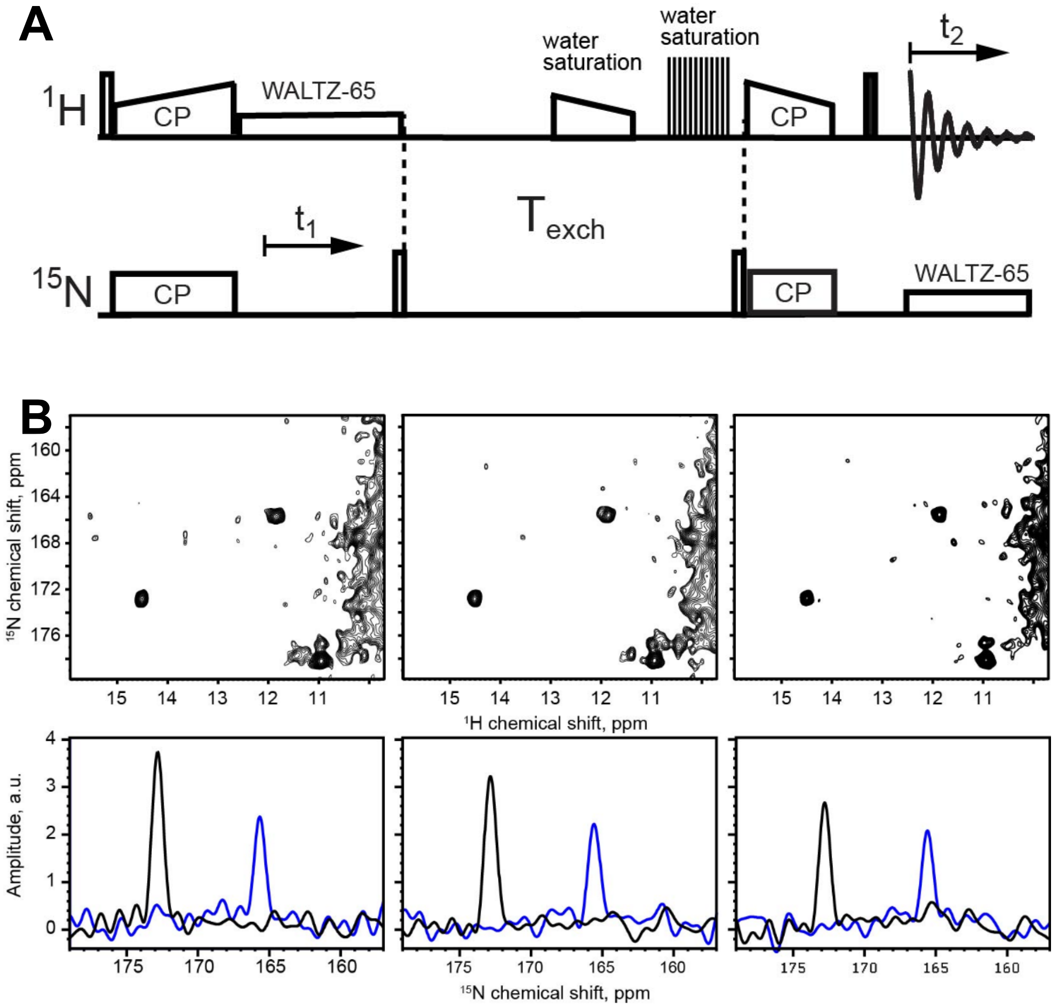

Figure S3: A) Two-dimensional pulse scheme employed to measure conformational exchange between two states of His-37 side chains. The representation of RF irradiation elements follows conventional notations, with open and filled bars representing 180° and 90° pulses, respectively, while the exchange period is denoted by T_{exch}. B) 2D HN correlations for histidine sidechain signal regions. The spectra obtained at 0, 2, and 10 seconds of exchange time are shown in the left, middle, and right column, respectively. The lower row represents 1D projections along the ^{15}N dimension for each exchange time for hydrogen-bonded (black) and free, i.e. no hydrogen bond (blue) state.

A

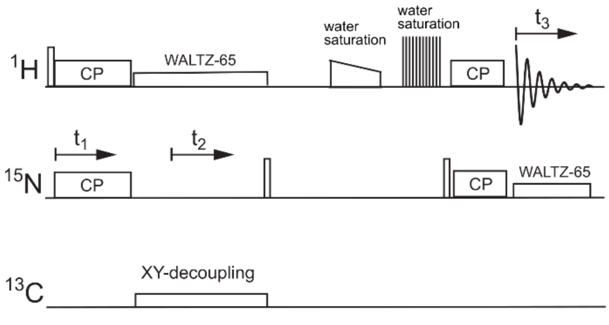

B

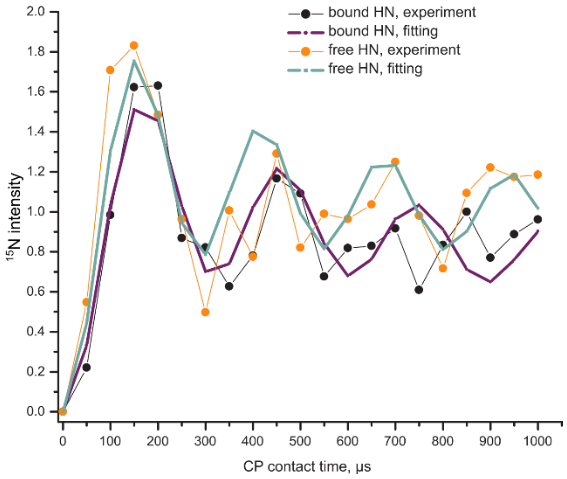

Figure S4: A) Pseudo 3D pulse scheme employed to measure ^{1}H-^{15}N dipolar couplings. The representation of RF irradiation elements follows conventional notations. B) Evolution of ^{15}N magnetization as a function of the CP contact time for free and bound H37 in M2. The experimental data are shown along with best-fit curves.

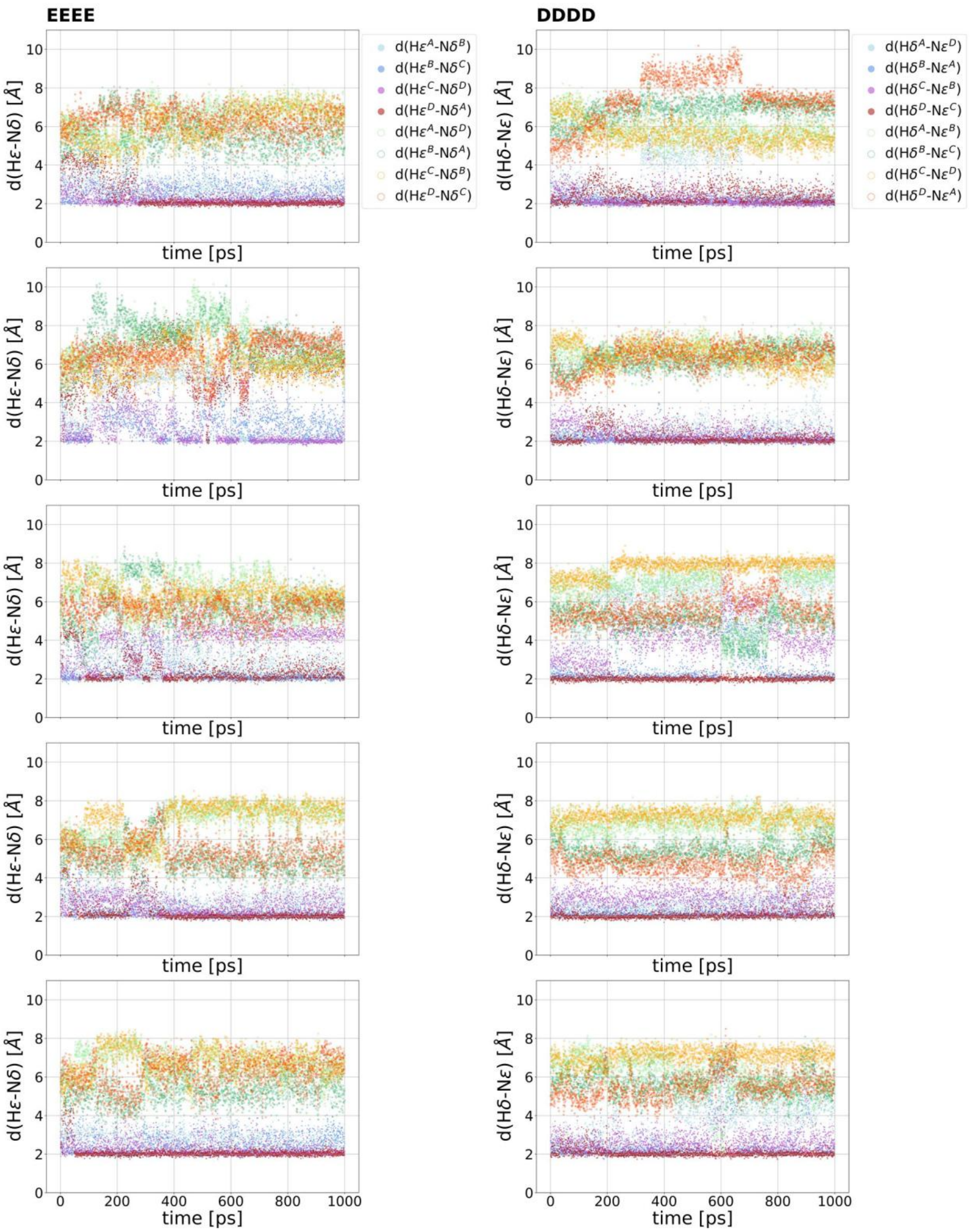

Figure S5: Hydrogen bond distances over time for five replicas of DFTB2+D QM/MM simulations; Interactions as indicated in the legend and defined in Figure S16.

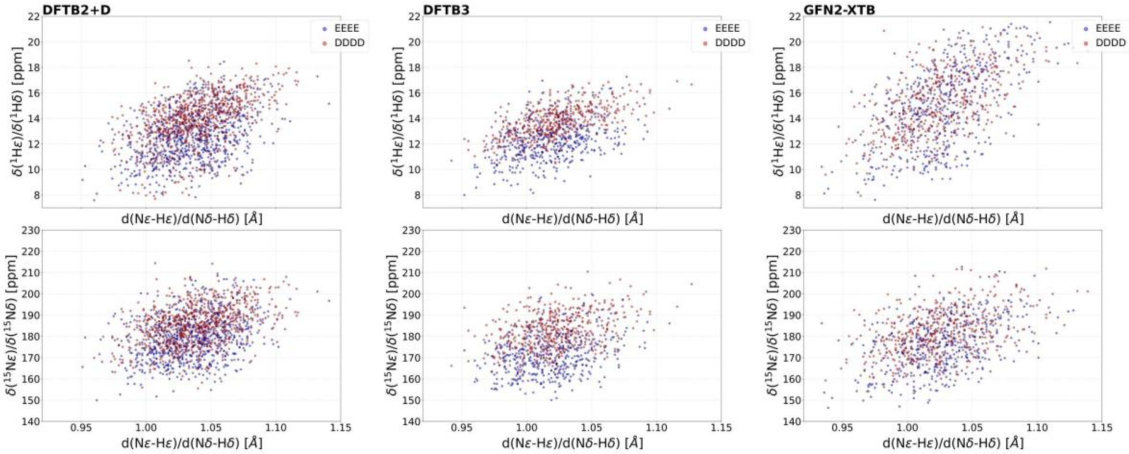

Figure S6: Predicted H37 chemical shift for structures extracted from DFTB2+D, DFTB3, and GFN2-XTB QM/MM models against bond distance in EEEE (d(Hε-Nε)) and DDDD (d(Hδ-Nδ)); chemical shifts in the top show ^{1}Hε, ^{1}Hδ, in the bottom line ^{15}Nε, ^{15}Nδ for EEEE and DDDD protonation states of histidine tetrad, respectively. Predicted chemical shifts on GIAO-MARIJ-cTPSS//pcSseg-2/pcSseg-1 level of theory.

Table S2: Bond length (Nε-Hε) averaged chemical shifts for EEEE model. Standard deviation of calculated shifts data is given as errors (± SD). Referenced against TMS (^{1}H), $NH_{3,liq}$ (^{15}N). Predicted chemical shifts on GIAO-MARIJ-cTPSS//pcSseg-2/pcSseg-1 level of theory.

EEEE		d(<0.98 Å)	d(<1.0 Å)	d(>1.0 Å)	d(>1.05 Å)
DFTB2	δ(^{1}Hε)	11.02 ± 1.57	11.18 ± 1.58	12.81 ± 1.92	13.45 ± 2.00
	δ(^{15}Nε)	173.1 ± 12.4	173.5 ± 10.1	180.3 ± 10.0	183.8 ± 10.3
DFTB3	δ(^{1}Hε)	10.77 ± 1.32	11.44 ± 1.29	12.62 ± 1.52	13.49 ± 1.59
	δ(^{15}Nε)	166.4 ± 7.3	167.9 ± 7.2	173.2 ± 9.7	178.5 ± 9.5
GFN2-XTB	δ(^{1}Hε)	11.35 ± 2.39	12.52 ± 2.52	15.42 ± 3.02	17.55 ± 2.64
	δ(^{15}Nε)	167.1 ± 12.3	169.1 ± 11.3	178.1 ± 11.6	184.1 ± 10.9

Table S3: Bond length (Nδ-Hδ) averaged chemical shifts for EEEE model. Standard deviation of calculated shifts data is given as errors (± SD). Referenced against TMS (^{1}H), $NH_{3,liq}$ (^{15}N). Predicted chemical shifts on GIAO-MARIJ-cTPSS//pcSseg-2/pcSseg-1 level of theory.

DDDD		d(<0.98 Å)	d(<1.0 Å)	d(>1.0 Å)	d(>1.05 Å)
DFTB2	δ(^{1}Hδ)	11.30 ± 1.99	12.22 ± 1.88	13.93 ± 1.89	14.95 ± 1.61
	δ(^{15}Nδ)	172.4 ± 5.3	178.1 ± 8.7	185.6 ± 9.6	189.6 ± 8.8
DFTB3	δ(^{1}Hδ)	12.16 ± 0.91	12.61 ± 0.95	13.99 ± 1.17	14.83 ± 1.03
	δ(^{15}Nδ)	176.6 ± 8.0	177.8 ± 8.1	183.3 ± 8.7	186.9 ± 9.1
GFN2-XTB	δ(^{1}Hδ)	12.38 ± 1.98	13.33 ± 2.02	15.85 ± 2.35	17.28 ± 2.08
	δ(^{15}Nδ)	172.6 ± 9.9	175.3 ± 9.7	183.7 ± 10.7	188.6 ± 10.2

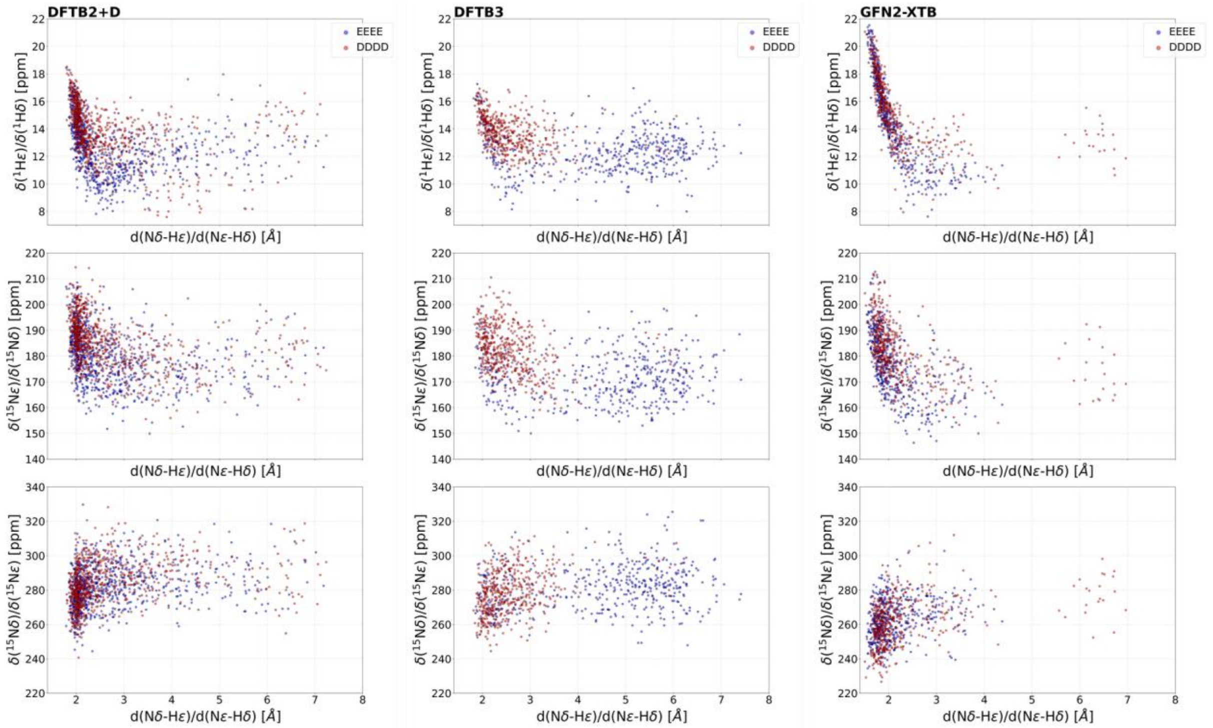

Figure S7: Predicted H37 chemical shift for structures extracted from DFTB2+D, DFTB3, and GFN2-XTB QM/MM models against smallest hydrogen bond distance in EEEE (d(Hε-Nδ)) and DDDD (d(Hδ-Nε)); chemical shifts in the top line show ^{1}Hε, ^{1}Hδ, in the middle line ^{15}Nε, ^{15}Nδ and the bottom line ^{15}Nδ, ^{15}Nε, for EEEE and DDDD protonation states of Histidine tetrad, respectively. Predicted chemical shifts on GIAO-MARIJ-cTPSS//pcSseg-2/pcSseg-1 level of theory.

Table S4: Hydrogen bond length (Hε-Nδ) averaged chemical shifts for EEEE model. Standard deviation of calculated shifts data is given as errors (± SD). Referenced against TMS (^{1}H), $NH_{3,liq}$ (^{15}N). Predicted chemical shifts on GIAO-MARIJ-cTPSS//pcSseg-2/pcSseg-1 level of theory.

EEEE		d(<2.3 Å)	d(<2.5 Å)	d(>2.5 Å)	d(>2.8 Å)
DFTB2	δ(^{1}Hε)	13.87 ± 1.70	13.42 ± 1.93	11.64 ± 1.44	11.85 ± 1.35
	δ(^{15}Nε)	183.9 ± 9.4	182.9 ± 9.6	175.5 ± 9.3	174.8 ± 8.8
	δ(^{15}Nδ)	278.0 ± 11.5	279.0 ± 11.8	285.7 ± 11.8	286.5 ± 11.8
DFTB3	δ(^{1}Hε)	13.66 ± 1.61	13.07 ± 1.86	12.13 ± 1.42	12.22 ± 1.37
	δ(^{15}Nε)	176.5 ± 9.3	174.7 ± 9.6	171.1 ± 9.3	171.4 ± 9.2
	δ(^{15}Nδ)	274.8 ± 11.9	276.2 ± 12.3	282.7 ± 13.0	283.1 ± 12.8
GFN2-XTB	δ(^{1}Hε)	16.21 ± 2.40	15.85 ± 2.64	10.73 ± 1.10	10.66 ± 1.05
	δ(^{15}Nε)	180.1 ± 10.4	179.0 ± 11.0	165.3 ± 9.9	165.4 ± 9.8
	δ(^{15}Nδ)	261.2 ± 11.4	261.5 ± 11.5	268.3 ± 10.4	268.4 ± 11.0

Table S5: Hydrogen bond length (Hδ-Nε) averaged NMR chemical shifts for DDDD model. Standard deviation of calculated shifts data is given as errors (± SD). Referenced against TMS (^{1}H), $NH_{3,liq}$ (^{15}N). NMR chemical shifts on GIAO-MARIJ-cTPSS//pcSseg-2/pcSseg-1 level of theory.

EEEE		d(<2.3A)	d(<2.5A))	d(>2.5 A)	d(>2.8A)
DFTB2	δ(^{1}Hε)	14.65±1.49	14.44±1.57	12.71±2.03	12.64±2.17
	δ(^{15}Nε)	189.2±8.1	188.7±8.3	178.8±8.6	177.9±8.5
	δ(^{15}Nδ)	279.0±11.8	279.9±12.0	290.0±12.0	289.9±11.6
DFTB3	δ(^{1}Hε)	14.35±1.10	14.1±1.17	13.21±1.19	13.15±1.15
	δ(^{15}Nε)	185.2±7.8	184.8±8.2	178.8±8.6	176.5±8.1
	δ(^{15}Nδ)	273.1±11.7	274.6±11.9	283±12.3	282.6±12.2
GFN2-XTB	δ(^{1}Hε)	16.16±2.13	15.92±2.29	12.58±1.33	12.55±1.34
	δ(^{15}Nε)	184.9±9.9	184.2±10.1	171.7±8.8	171.2±8.6
	δ(^{15}Nδ)	258.3±12.4	259.1±12.8	271.1±13.2	271.4±13.2

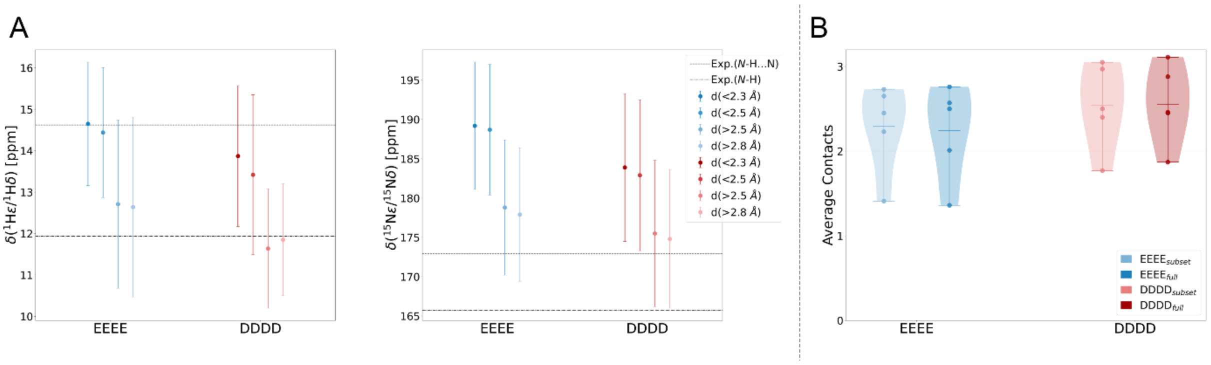

Figure S8: A) Distance averaged NMR chemical shifts; ^{1}Hε and ^{1}Hδ on the left, ^{15}Nε and ^{15}Nδ on the right for models as indicated in the bottom. (B) Average contacts per frame for subsets (40 frames) as shown in (A, left) and full trajectory (2000 frames).

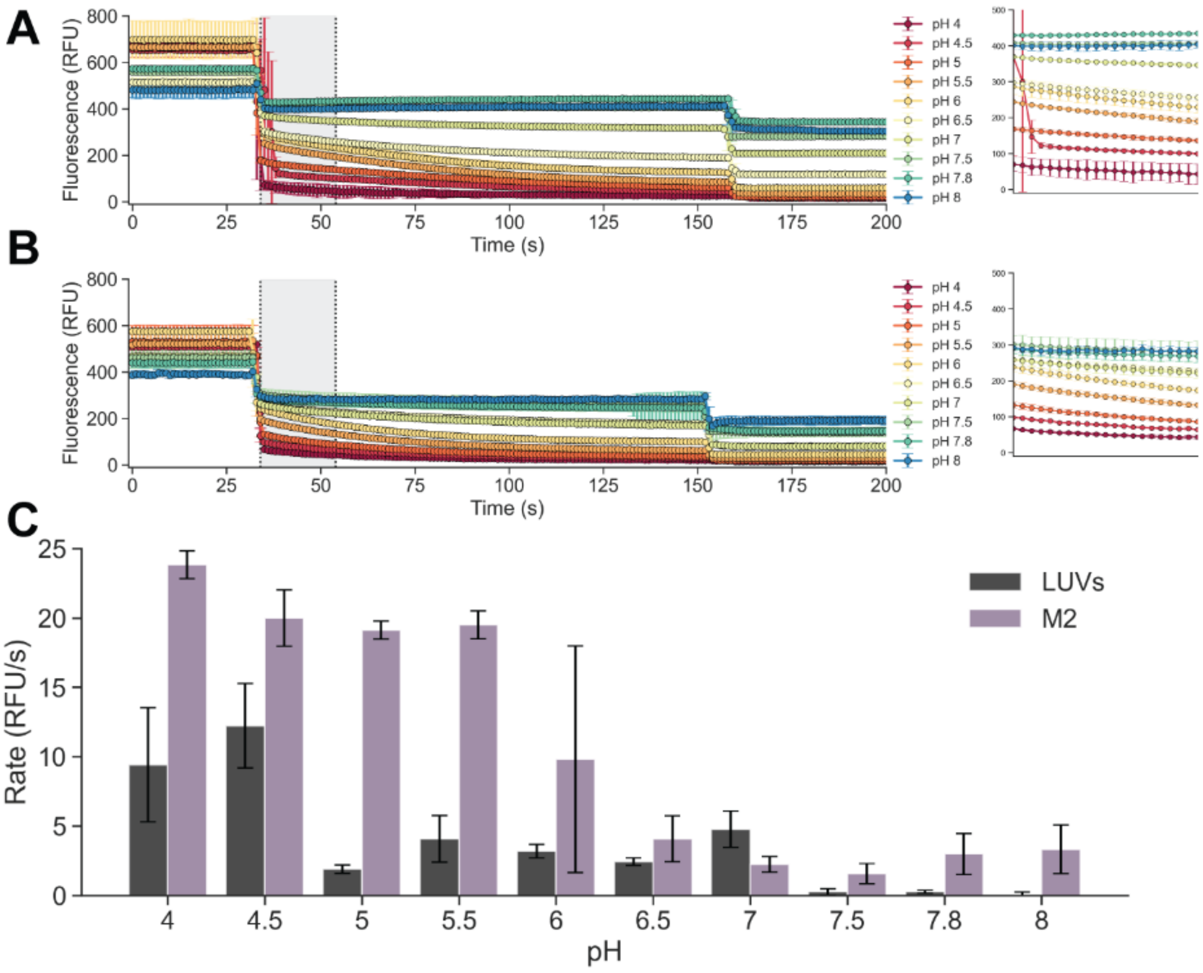

Figure S9: Functional assay data of liposomal proton flux assay, showing fluorescence intensity at 510 nm for *E. coli* LUVs (A) and M2 (B) containing samples. Liposomes were incubated for 30 seconds prior to initiation with defined pH buffer, left to incubate for 120 s before addition of triton for baseline measurement. Grey shaded region denotes time period taken for rate calculation by linear regression after initial decrease due to dilution (A and B insert, right). (C) Average rate for both LUVs (black) and M2 (purple) samples, across stated pH range. Error denotes technical triplicate.

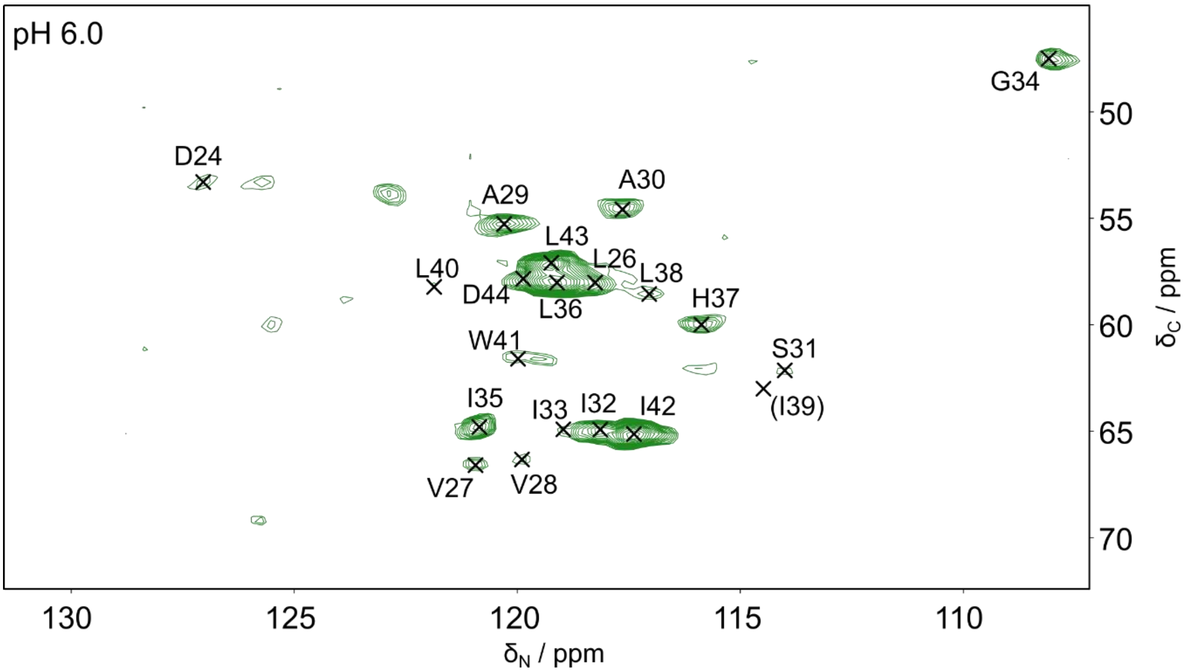

Figure S10: NC-projection of (H)CαNH-experiment of the M2 sample reconstituted in a pH 6.0 environment. Assigned resonances are labeled.

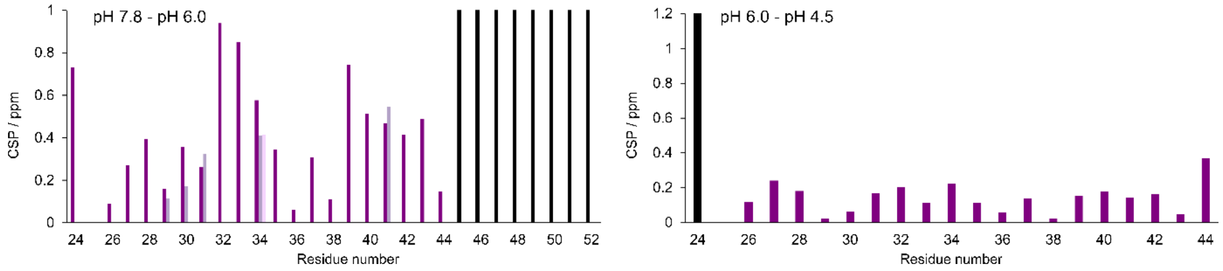

Figure S11: Chemical shift perturbation plot of the pH 6.0 sample compared to the pH 7.8 sample (left) and pH 6.0 compared to pH 4.5 (right). Residues showing multiple resonances at pH 7.8 are displayed with multiple bars, showing the different CSP values for each peak. Residues with disappearing resonances are displayed as black bars.

Table S6: Results from the TALOS+ analysis of the assigned chemical shifts for pH 4.5 and 7.8, with Phi and Psi being the predicted backbone torsion angles, dPhi and dPsi the standard deviations of the calculated angles, and ss being the predicted secondary structure (c=coil, sequence based; L=coil, chemical shift based; h=helix, sequence based; H=helix, chemical shift based).

		pH 7.8					pH 4.5				
Nr.	Res.	Phi	Psi	dPhi	dPsi	ss	Phi	Psi	dPhi	dPsi	ss
24	D	9999	9999	0	0	c					
25	P	-63.76	-23.53	9.18	10.39	H	9999	9999	0	0	c
26	L	-62.71	-36.35	4.90	6.43	H	-60.90	-36.62	4.26	5.25	H
27	V	-68.46	-39.15	4.98	5.32	H	-64.17	-38.65	5.42	5.29	H
28	V	-65.63	-42.66	3.34	3.99	H	-66.77	-42.92	3.56	3.22	H
29	A	-62.95	-41.49	4.32	4.25	H	-63.20	-43.35	4.33	4.75	H
30	A	-64.09	-41.09	3.25	4.34	H	-63.27	-41.35	3.45	3.50	H
31	S	-65.40	-42.39	2.99	3.85	H	-64.68	-41.00	3.50	3.61	H
32	I	-64.62	-42.24	2.85	4.80	H	-64.77	-44.05	3.97	3.67	H
33	I	-64.74	-39.75	3.25	4.04	H	-63.90	-38.44	4.32	5.13	H
34	G	-62.78	-41.55	3.72	3.87	H	-63.86	-41.55	2.94	4.11	H
35	I	-64.41	-44.68	3.70	3.98	H	-65.77	-43.06	3.34	3.50	H
36	L	-65.27	-39.09	3.23	5.24	H	-64.05	-40.61	3.13	5.12	H
37	H	-62.98	-42.33	4.45	4.44	H	-63.95	-41.74	3.72	5.30	H
38	L	-63.53	-44.88	3.46	3.08	H	-64.25	-44.80	3.25	3.19	H
39	I	-63.01	-42.77	4.24	3.87	H	-63.50	-42.99	2.94	5.08	H
40	L	-65.30	-40.88	4.06	4.90	H	-65.51	-41.05	4.64	5.08	H
41	W	-62.69	-42.75	3.30	4.37	H	-62.39	-44.42	3.34	3.41	H
42	I	-63.85	-44.84	4.18	3.24	H	-63.99	-44.18	4.11	4.43	H
43	L	-64.77	-40.46	3.70	4.35	H	-61.77	-38.03	4.16	5.14	H
44	D	-63.34	-40.77	4.46	6.01	H	-64.26	-23.81	5.74	8.96	H
45	R	-70.04	-34.56	4.71	6.00	H	9999	9999	0	0	h
46	L	-71.54	-27.69	8.97	6.92	H					
47	F	-104.40	1.90	8.03	10.81	H					
48	F	-61.34	-42.36	6.10	6.27	L					
49	K	-66.00	-38.04	6.10	6.85	L					
50	S	-67.43	-37.72	5.55	6.37	H					
51	I	-62.38	-36.29	5.79	5.34	H					
52	Y	9999	9999	0	0	L					

Table S7: Overview of simulations. Perimeter.

Name	perimeter V27 / Å	perimeter H37 / Å	perimeter W41 / Å	perimeter D44 / Å
2L0J	32.93	36.20	41.14	56.35
DDDD	31.08 ± 0.06	35.44 ± 0.14	38.83 ± 1.11	50.06 ± 0.81
EEEE	30.11 ± 1.44	35.72 ± 0.80	39.55 ± 1.23	49.93 ± 1.19
PDPD	30.97 ± 0.23	37.05 ± 0.60	44.32 ± 1.43	55.50 ± 2.82
PEPE	31.29 ± 0.17	36.84 ± 0.96	43.14 ± 1.67	54.54 ± 3.39
PPDD	31.22 ± 0.08	36.88 ± 0.45	43.19 ± 1.49	53.84 ± 2.33
PPEE	31.16 ± 0.26	37.71 ± 1.86	44.42 ± 3.38	55.06 ± 5.12
PPPE*	30.51 ± 0.04	42.25 ± 1.53	53.64 ± 4.29	68.92 ± 5.21
PPPP*	30.65 ± 0.30	45.25 ± 1.54	61.21 ± 3.57	78.17 ± 4.28

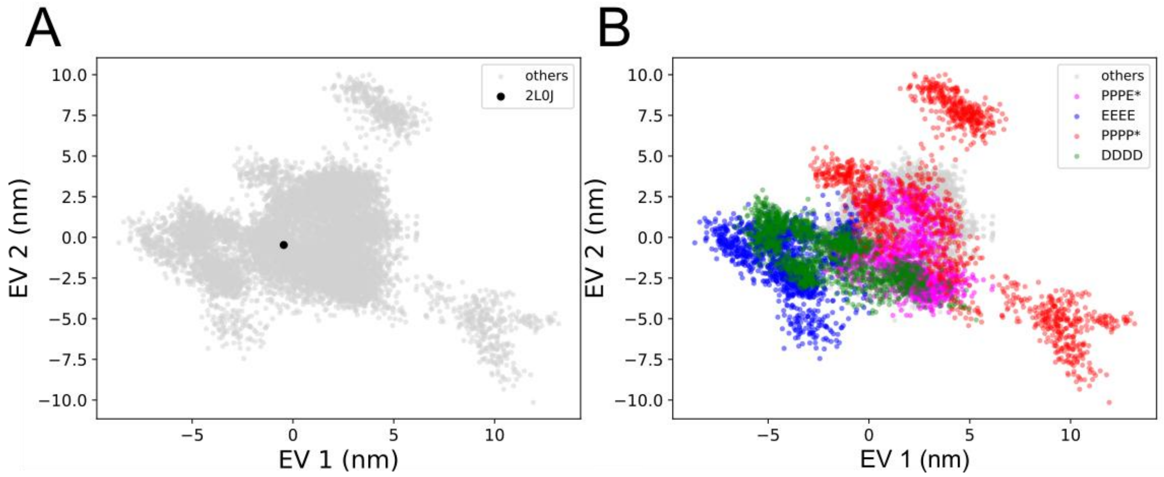

Figure S12: (A) Projection of the first and second eigenvectors (EV) onto the eigenvector space defined by the EEEE and PPPP* models (grey). The black dot represents the crystal structure (PDB: 2L0J). EV1 is mainly describing the opening dynamics of M2. The projection shows that more open and more closed conformations are sampled compared to the crystal structure. (B) Projection of EV1 and EV2 of the EEEE (blue), DDDD (green), PPPE* (magenta), and PPPP* (red) models onto the eigenvector space defined by EEEE and PPPP* models. The color code corresponds to figure 5 A and B. Grey indicates the other models (PDPD, PEPE, PPDD, and PPEE). It is clearly visible that the high degree of H37 protonation in PPPP* and PPPE* leads to higher EV1 values, while EEEE and DDDD result in lower EV1 values corresponding to a closed pore.

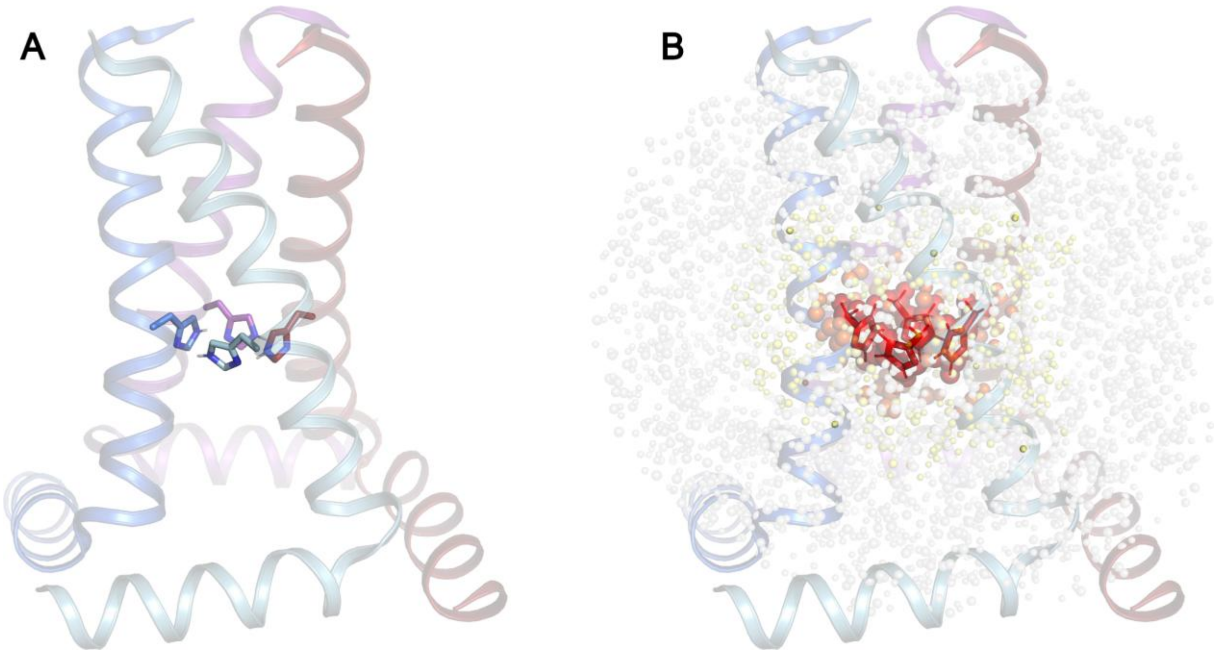

Figure S13: (A) M2 Viroporin chains A-D with highlighted H37 tetrad; (B) Exemplary depiction of the QM NMR model with highlighted qm shells for adapted locally dense basis-set approach (red highlights the chosen QM atoms (pcSseg-2 basis), orange the first shell QM atoms (pcSseg-2 basis), yellow the second shell QM atoms (pcSseg-1) basis set, and in grey the point charge embedding is shown).

Additional QM/MM calculations

To further validate the QM/MM simulations comprised in the main text, i.e. characterize the bonding patterns in more detail, we here present data for additional QM/MM simulations on DFTB3, GNF2-XTB, and DFTB2 (excluding the dispersion correction) level of theory.

In these simulations, fewer average contacts are found in DFTB3 and DFTB2 models without dispersion correction, whereas, for GFN2-XTB simulations average contacts are increased somewhat in DDDD, yet significantly in EEEE in comparison to DFTB2+D, and now exceed three contacts on average in both models (Figures S14 and S15, as well as Table S8). Nevertheless, comparing the NMR chemical shifts against the hydrogen bonding distances of DFTB3 and GFN2-XTB simulations (Figure S6 and Tables S2 and S3) with the DFTB2+D data of Figure 2B, consistently in all QM models a similar almost linear deshielding is associated with shorter hydrogen bonds. In GFN2-XTB the diffuse interactions with longer hydrogen bond distances are less pronounced, and as a result the distance average shielding differences increase.

Within the assigned error margins, however, reasonable agreement of ^{1}H and ^{15}N experimental and computational shift differences is observed in all models (Tables S2 and S3).

The differing averaged contacts with various employed tight binding models appear to be the result of the different parameters, and importantly, treatment of non-covalent interactions, i.e. hydrogen bonding within the HIS37 tetrad: In DFTB2 reduced error margins were reported for hydrogen-bonding base pairs employing the dispersion-correction[1], which for our simulations

appears to result in more average contacts in EEEE. However, even slightly increased average contacts are observed without dispersion for DDDD. At the present stage, we cannot clearly determine whether this is due to the small number of replicas, error compensation or other effects of the approximate model Hamiltonian used in DFTB2.

For GFN2-XTB, dispersion is built into the model, and increased average contacts were observed for both models. Previously, for DFTB3 inclusion of dispersion was found to decrease underbinding in hydrogen bonded systems[2], however, no dispersion model for DFTB3 is included in the AMBER program code, hence was at this stage not tested. We also note in passing that both DFTB2 and DFTB3 models slightly overestimated bond length in CHNO containing compounds (hence underestimate interaction strength)[3], regardless of dispersion correction[4]. In contrast, a reversed trend is observed for GFN2-XTB, which appears to align with the more deshielded bond-length averaged ^{1}H NMR shifts (see Tables S2 and S3).

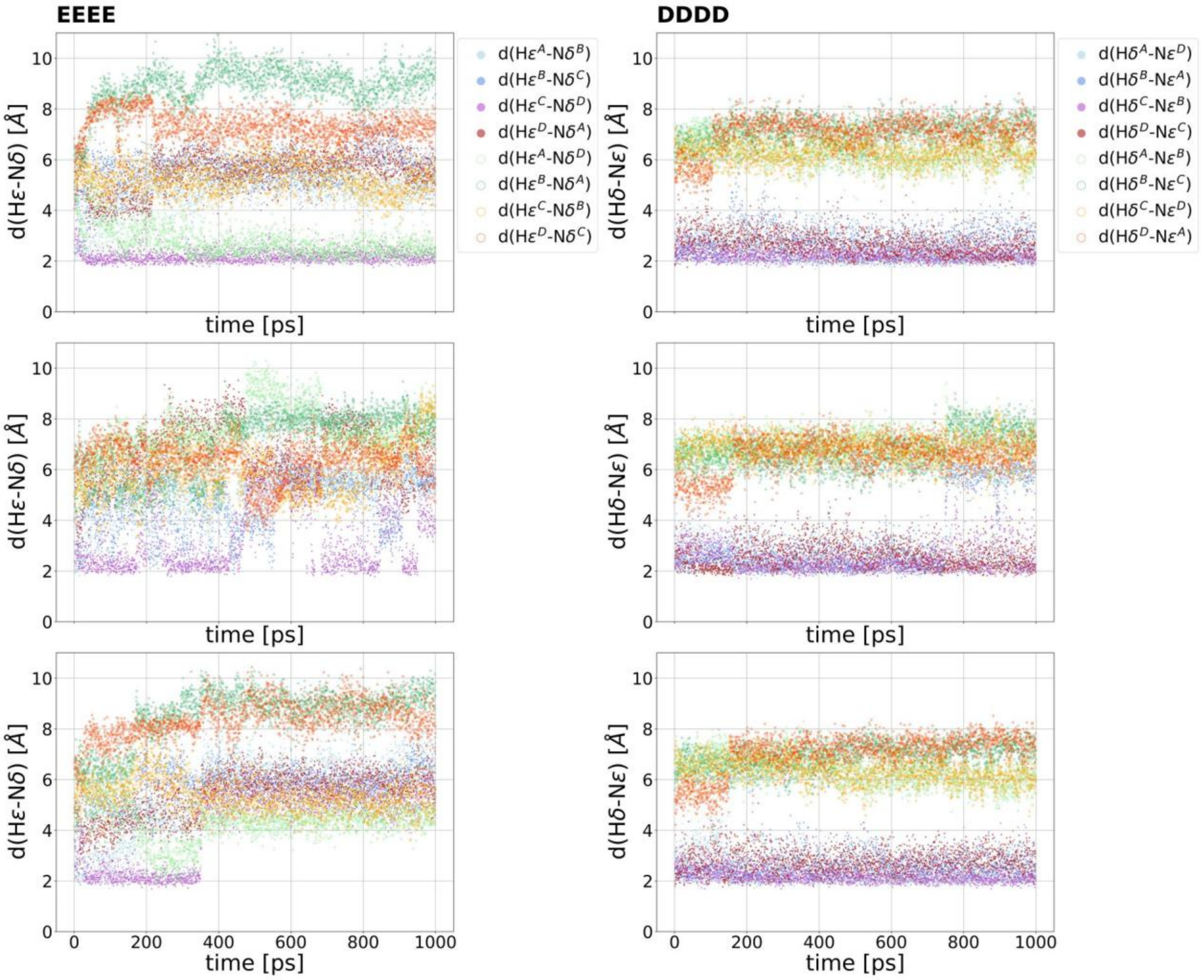

Figure S14: Hydrogen bond distances over time for five replicas of DFTB3 QM/MM simulations; Interactions as indicated in the legend and defined in Figure S16.

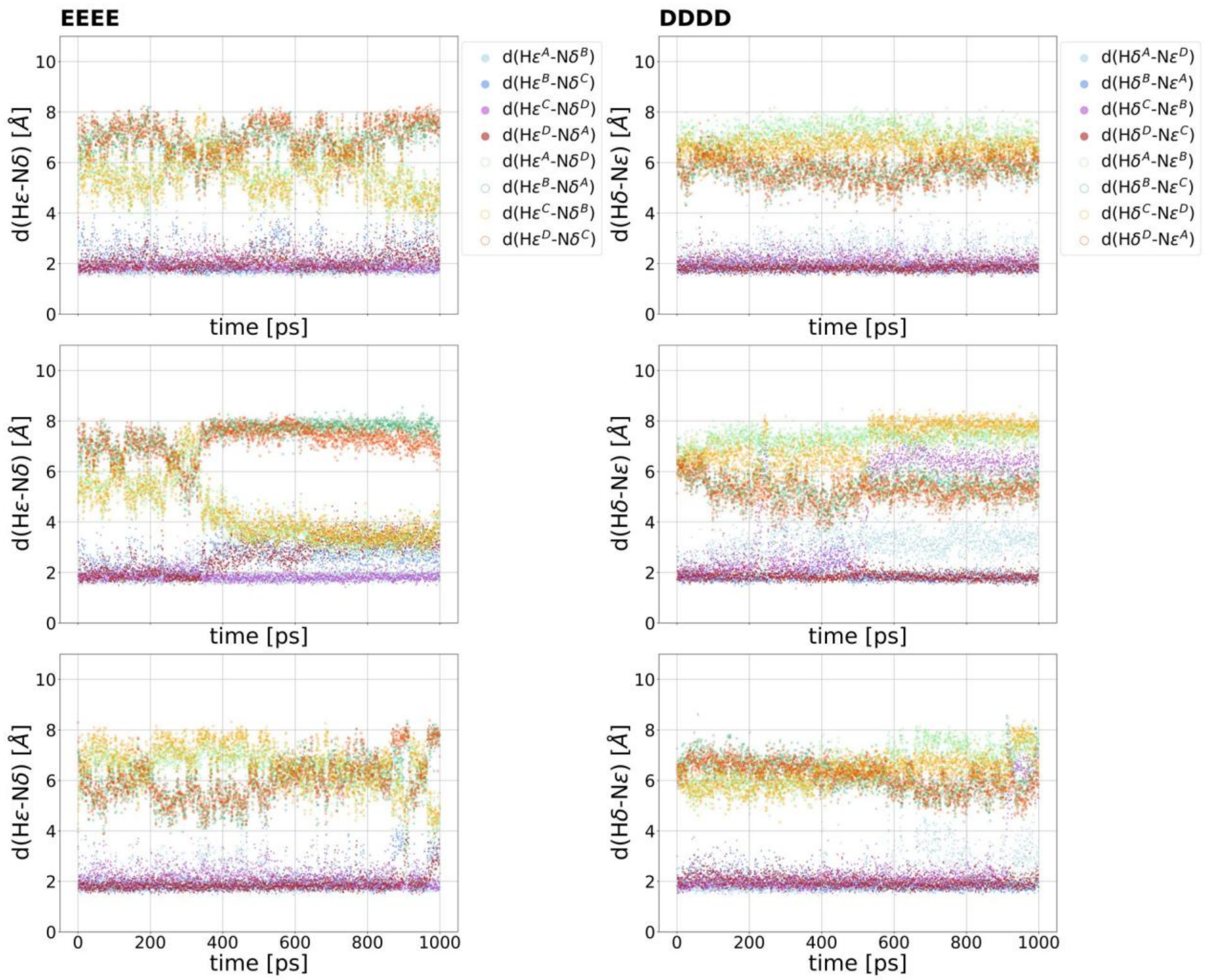

Figure S15: Hydrogen bond distances over time for five replicas of GFN2-XTB QM/MM simulations; Interactions as indicated in the legend and defined in Figure S16.

Table S8: Average contacts (d<2.5 Å) per frame in different QM models for QM/MM simulations within the His37 tetrad; data shown for individual replicas and overall average in models EEEE and DDDD. Full trajectory results consider potential clockwise and counterclockwise contacts; for subsets we consider contacts exemplified in Figure 3.

		Replica	1	2	3	4	5	Avg.
DFTB2+D	EEEE	Subset	2.73	1.41	2.23	2.45	2.65	2.29
		Full Tra.	2.50	1.36	2.01	2.57	2.76	2.24
	DDDD	Subset	2.97	3.05	1.77	2.40	2.50	2.54
		Full Tra.	2.88	3.11	1.87	2.45	2.46	2.55
DFTB2	EEEE	Full Tra.	2.43	1.19	0.30			1.31
	DDDD	Full Traj	2.50	2.93	3.23			2.89
DFTB3	EEEE	Full Tra.	1.34	0.48	0.38			0.73
	DDDD	Full Tra.	2.25	2.21	2.30			2.25
GFN2-XTB	EEEE	Full Tra.	3.49	2.78	3.52			3.26
	DDDD	Full Tra.	3.55	2.46	3.51			3.17

Table S9: Adapted dispersion correction parameters for DFTB2.

O	0.560	0.560	0.560	0.560	3.8	3.8	3.8	3.8	3.15
N	1.030	1.030	1.090	1.090	3.8	3.8	3.8	3.8	2.82
C	1.382	1.382	1.382	1.064	3.8	3.8	3.8	3.8	2.5
H	0.386	0.386	0.386	0.386	3.5	3.5	3.5	3.5	0.8
P	1.6	1.6	1.6	1.6	4.7	4.7	4.7	4.7	4.5
S	3.0	3.0	3.0	3.0	4.7	4.7	4.7	4.7	4.8

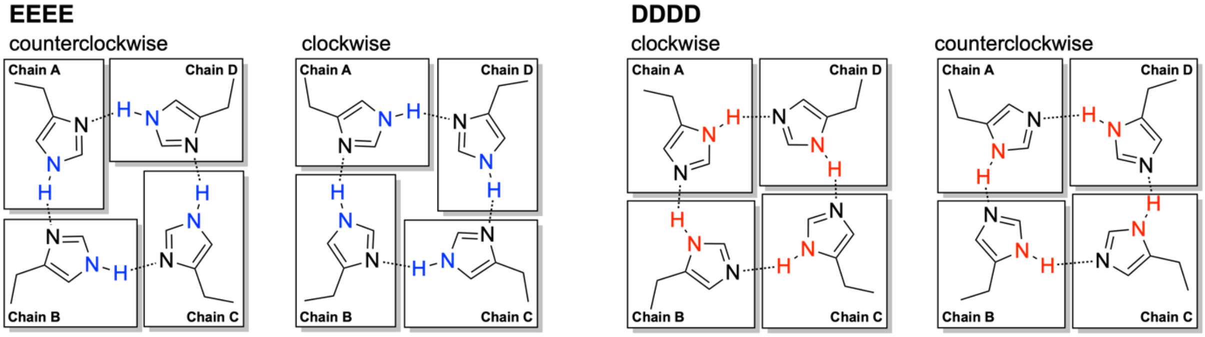

Figure S16: Definition of clockwise and counterclockwise hydrogen bonds in EEEE and DDDD, respectively, as depicted in legends of Figures S5, S14 and S15.

References:

1. Elstner, M.; Hobza, P.; Frauenheim, T.; Suhai, S.; Kaxiras, E. Hydrogen bonding and stacking interactions of nucleic acid base pairs: A density-functional-theory based treatment. *J Chem Phys* **2001**, *114,* 5149–5155.
2. Brandenburg, J. G.; Grimme, S. Accurate Modeling of Organic Molecular Crystals by Dispersion-Corrected Density Functional Tight Binding (DFTB). *The Journal of Physical Chemistry Letters* **2014**, *5(11)*, 1785–1789.
3. Gaus, M.; Cui, Q.; Elstner, M. DFTB3: Extension of the self-consistent-charge density-functional tight-binding method (SCC-DFTB). *J Chem Theory Comput* **2011**, *7,* 931–948.
4. Bannwarth, C.; Ehlert, S.; Grimme, S. GFN2-xTB - An Accurate and Broadly Parametrized Self-Consistent Tight-Binding Quantum Chemical Method with Multipole Electrostatics and Density-Dependent Dispersion Contributions. *J Chem Theory Comput* **2019**, *15*, 1652–1671.
